# Strand-Swapped SH3 Domain Dimer with Superoxide Dismutase Activity

**DOI:** 10.1021/acscentsci.4c01347

**Published:** 2025-01-10

**Authors:** Florian R. Häge, Merlin Schwan, Marcos Rafael Conde González, Jonas Huber, Stefan Germer, Matilde Macrì, Jürgen Kopp, Irmgard Sinning, Franziska Thomas

**Affiliations:** † Institute of Organic Chemistry, Heidelberg University, Im Neuenheimer Feld 270, 69120 Heidelberg, Germany; ‡ Biochemistry Center, Heidelberg University, Im Neuenheimer Feld 328, 69120 Heidelberg, Germany; § Max Planck School Matter to Life, Jahnstraße 29, 69120 Heidelberg, Germany

## Abstract

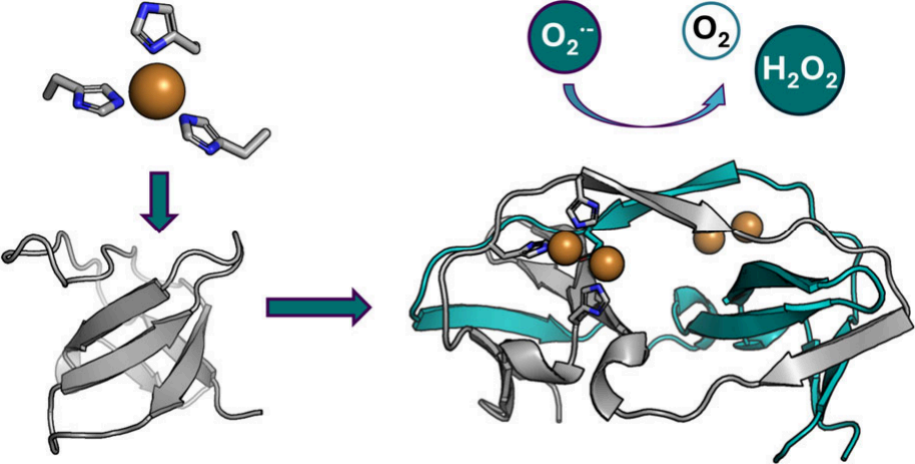

The design of metalloproteins allows us to better understand metal complexation in proteins and the resulting function. In this study, we incorporated a Cu^2+^-binding site into a natural protein domain, the 58 amino acid c-Crk-SH3, to create a miniaturized superoxide dismutase model, termed SO1. The resulting low complexity metalloprotein was characterized for structure and function by circular dichroism and UV spectroscopy as well as EPR spectroscopy and X-ray crystallography. The miniprotein was found to be a strand-swapped dimer with an unusual coupled binuclear Type 2-like copper center in each protomer. SO1-Cu was found to be SOD-active with an activity only 1 order of magnitude lower than that of natural SOD enzymes and 1 to 2 orders of magnitude higher than that of other low-complexity SOD protein models of similar size. This serendipitous design provides us with a new structural template for future designs of binuclear metalloproteins with different metal ions and functions.

## Abstract 2

The catalytic copper site of human Cu,Zn Superoxide Dismutase 1 was grafted onto the c-Crk SH3 domain, resulting in a strand-swapped SH3 domain dimer with remarkable superoxide dismutase activity.

## Introduction

Metalloproteins are ubiquitous in nature, accounting for 25–50% of all proteins and 50% of all enzymes.^1,2^ One way to study the effect of metal ions on protein structure and function is to design metalloproteins with reduced size and structural complexity compared to their natural counterparts.^3,4^ This improves the understanding of the interaction of metal ions with the coordinating amino acids, the influence of the second coordination sphere on the function of a protein or the balance between the embedding of a metal ion in a coordinating protein scaffold and its accessibility to the environment.

*De novo* design of metalloproteins is the ideal approach to gain improved understanding of the influence of a coordinated metal ion on a protein structure and function.^3^ Metalloproteins have been designed to exhibit hydrolytic, reductive, oxidative and electron transfer activities.^5^ The design was not limited to mononuclear metal binding sites, but binuclear metal centers were also introduced. Of particular note is the binuclear Due-Ferri system, members of which exhibit catalytic activity in the oxidation of organic substrates such as 3,5-di-*tert*-butylcatechol, *p*-aminophenol and *p*-phenylenediamine.^6−8^

However, *de novo* design is only possible if a protein scaffold can be designed with a high degree of predictability, which usually correlates with very well understood sequence-structure relationships. It is therefore not surprising that most globular *de novo* metalloproteins are built on α-helical scaffolds,^9,10^ which limits the explored structural space. Although impressive successes have been achieved in the design of catalytic metal-binding amyloids,^11,12^ very few catalytic globular model metalloprotein systems have been described based on the β-sheet secondary structure, mainly due to a lack of understanding of sequence-structure relationships.^13^ However, in these cases, the engineering of natural, robustly folded protein motifs is a proven means of obtaining miniaturized models of natural metalloproteins.^14^ Examples include β-hairpin heme complexes with redox activity.^15−18^ In addition, β-sheet miniproteins, β-hairpins and WW domains have been reported to bind nickel, copper and zinc ions, but no catalytic activity has yet been demonstrated.^19−21^

In addition to the exploration of small protein folding motifs in the design of metalloprotein models, considerable success has also been achieved with dimerization approaches in which protein domains are either covalently linked by a disulfide bridge, the so-called MASCoT (Metal Active Sites through Covalent Tethering) approach,^22^ or by engineering metal-binding sites in domain-swapped proteins.^23,24^ Strand or domain swapping describes a process in which part of the protein structure is exchanged between identical protein monomers to form dimers or higher oligomers without altering the secondary structure of the monomer units.^25^ The key to this dynamic process is the opening of a hinge loop, which leads to the association and repackaging of the two protomer units. One highly interesting approach toward artificial metalloproteins involves the engineering of the metal-binding site at the hinge regions, which allows the binding of metal ions only when the protein in question is in the domain swap state.^26^

To explore β-sheet scaffolds in the context of catalytically active metalloprotein design, we turned to the SH3 domain (SH3 – Src homology 3).^27^ SH3 domains, which are protein-binding domains, consist of five to six β-strands arranged as two antiparallel β-sheets and are about 60 amino acids long, making them a suitable protein scaffold for the design of model metalloproteins. Our aim was to develop a catalytically active scaffold with superoxide dismutase (SOD) activity. SOD proteins disproportionate superoxide into molecular oxygen and hydrogen peroxide and are part of the enzymatic machinery that protects the cell from oxidative stress.^28,29^ They can be divided into three families according to the nature of metal ion in the active center: Ni SODs, Fe,Mn SODs and Cu,Zn SODs, the latter being the most abundant in eukaryotic cells.^30,31^ Because of their antioxidant properties, SOD mimics have been sought for use as antioxidant therapeutics.^32−35^ Many low molecular weight complexes have been developed that exhibit impressive catalytic efficiencies, in some cases only one order of magnitude lower than the natural enzyme.^33,36−38^ However, for a better understanding of the structure–function relationships of SODs, the use of a low-complexity protein scaffold into which SOD activity is engineered is more appropriate, as it better simulates the natural protein environment. In this context, α-helical coiled-coil scaffolds with manganese and copper centers have been explored, which are low in complexity but show only moderate catalytic efficiencies, four to three orders of magnitude lower than those of natural SODs.^39,40^ A top-down designed thioredoxin-based biomimetic protein was also investigated, but displayed a catalytic efficiency similar to the coiled-coil biomimetic SODs.^41,42^

The SH3 domain displays a higher structural similarity to Cu,Zn SODs, thus should be a more suitable scaffold to mimic its activity.^27^ Using the Rosetta design suite, we grafted the copper-binding site of human Cu,Zn SOD1 (hSOD1) onto the c-Crk SH3 domain and obtained a metalloprotein that binds to first-row transition metal ions Mn^2+^, Co^2+^, Ni^2+^, Cu^2+^ and Zn^2+^ with binding dissociation constants in the micromolar to nanomolar range. The modified model protein displayed SOD activity that was reduced by a factor of only 40 to 70 compared to natural SODs. However, X-ray crystallography supported by CD spectroscopy, EPR spectroscopy and Cu^2+^ titration experiments showed that the engineering of the c-Crk SH3 domain resulted in a strand-swapped SH3 domain dimer with two binuclear copper centers in the holo state. The complex is a rare example of an engineered strand-swapped metalloprotein for two reasons: 1) the metal-binding site is formed at the hinge region, making SO1 only the second example of such a system described in the literature;^24,26^ 2) SO1 displays an unusual coupled binuclear Type 2-like copper protein unrelated to the Type 3 copper centers of e.g. tyrosinase, catechol oxidase and laccase.

## Results

### Design Concept

**Figure 1:**
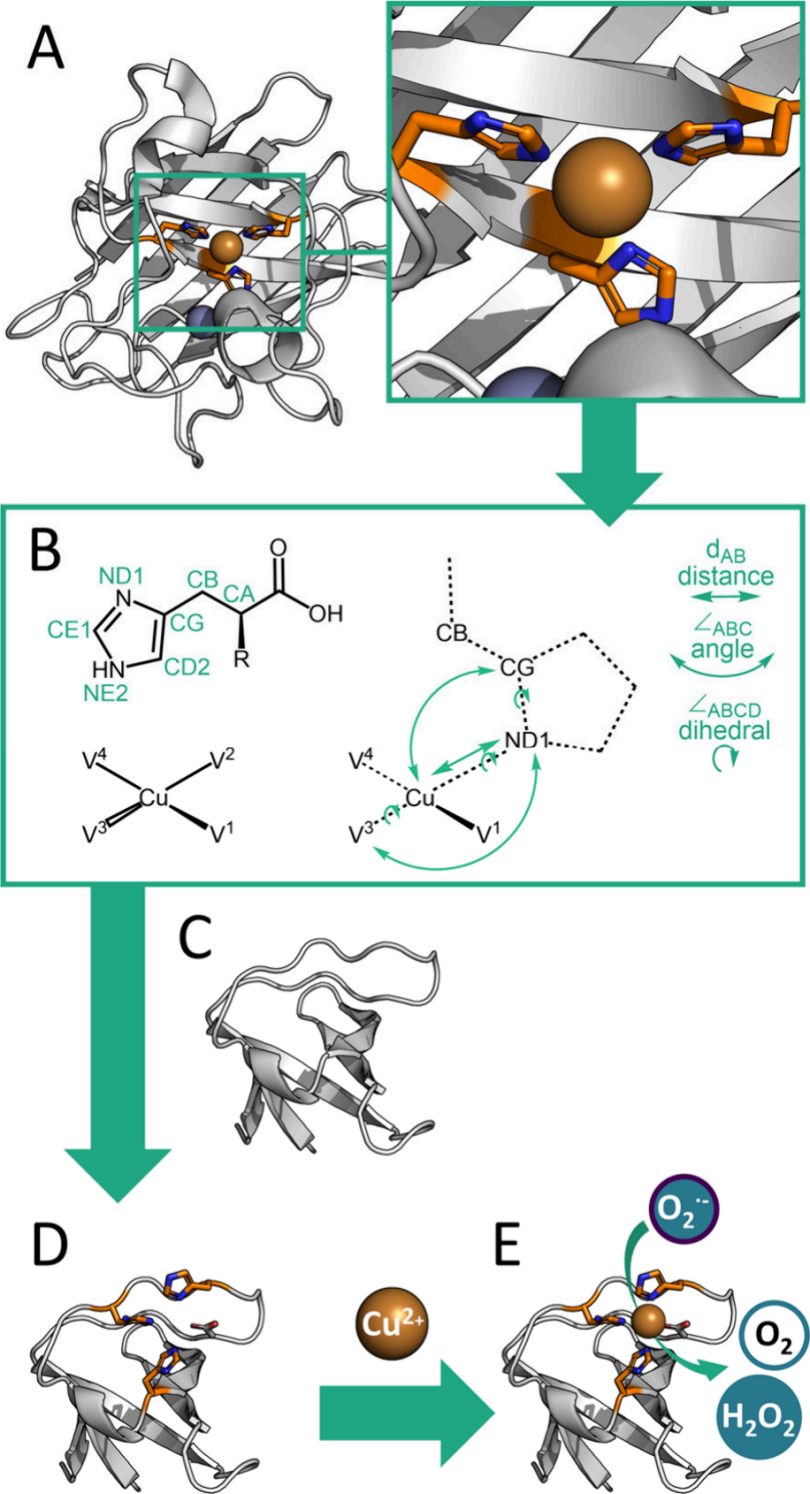
Design approach of an SOD minienzyme. (A) X-ray crystal structure of hSOD1 used as a design template (PDB 3CQP) with zoom of the active center and (B) extracted geometric parameters; (C) the N-terminal c-Crk SH3 domain (PDB 1CKB) as model scaffold, and (D) the model of the SO1 miniprotein designed with the Rosetta3 modeling suite and (E) model of the active SO1-Cu minienzyme (for details see the SI).

We chose hSOD1 as a natural model for the design of a miniaturized protein with superoxide dismutase activity (PDB 3CQP).^43^ hSOD1 is a homodimer with each subunit describing an antiparallel eight-stranded β-barrel structure arranged in a Greek key motif. Both subunits contain a catalytically active Cu,Zn site balanced between two loops, where the metal ions are coordinated tetrahedrally, with the structure-stabilizing Zn^2+^ complexed by three histidine residues (H63, H71, H80) and one aspartate residue (D83), and the catalytic Cu^2+^ complexed by four His (H46, H48, H63, H120). The two metal centers are bridged by H63 in the oxidized Cu^2+^ state (Figure 1A, Figure S1).

**Figure 2:**
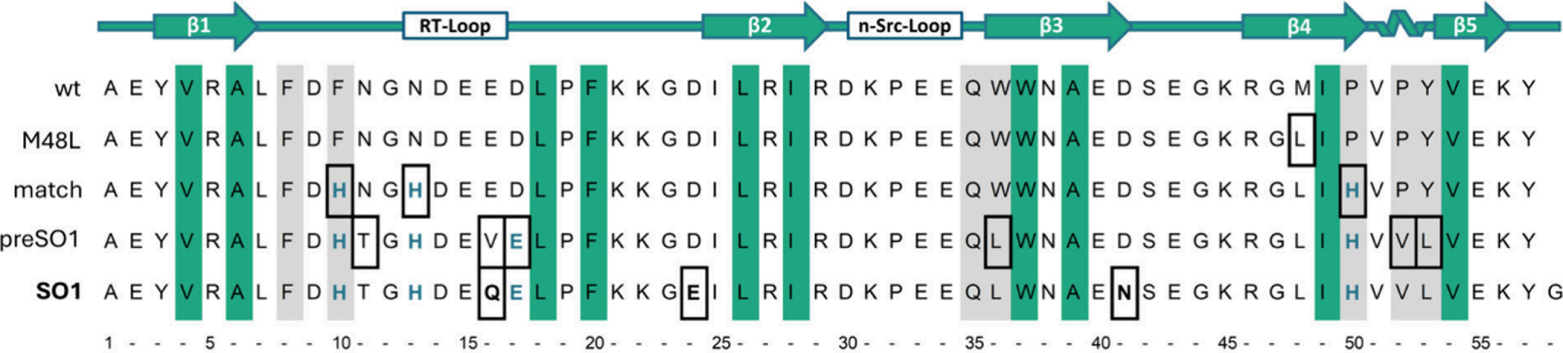
Sequence design of SO1. Schematic representation of the secondary structure motifs of the natural c-Crk SH3 domain (top, arrows indicate β-sheet and helix indicates a helical segment) and sequence outputs in the design of SO1 (bottom). The conserved positions of the hydrophobic core are highlighted in green and the conserved positions of the peptide binding site in gray. New mutations are boxed, Cu^2+^ coordinating amino acids are depicted in teal. wt: Sequence of the c-Crk-SH3. M48L: Sequence of c-Crk-SH3 in which the oxidation-prone amino acid Met at position 48 has been replaced by Leu. match: Sequence of output model obtained by the Match application of Rosetta3 indicating the three His residues (teal) for coordination to Cu^2+^. preSO1: Sequence of the lowest energy model obtained by optimizing the match sequence with Rosetta Design. The sequence positions modified by Rosetta Design are boxed, and the coordinating residues are shown in teal. SO1: Final sequence of the designed SOD active SH3 domain. Val16 has been replaced by Gln to achieve better similarity to the original wildtype loop. Asp residues have been replaced by Glu or Asn residues to avoid aspartimide formation during chemical synthesis.

Since Zn^2+^ in hSOD1 mainly plays a structure-stabilizing role, we decided to develop a Cu^2+^-only SOD minienzyme.^30,44^ Previous peptide/protein-based model systems, such as a *de novo* designed 100 amino acid α-helical protein model, have shown that the structure around the Cu^2+^ coordination site does not require a β-sheet character to yield a moderately active SOD model.^39,40^ However, in order to obtain an SOD model with improved activity, we decided to use a β-sheet scaffold as the starting point for our design because it is more similar to the scaffold of natural Cu,Zn SODs. We also envisaged a scaffold that is considered a mini-protein because it is less than 100 amino acids in size. The N-terminal SH3 domain of c-Crk (c-Crk-SH3), which has been shown to be accessible by chemical synthesis, was therefore used for a top-down design approach.^27,45,46^ Since oxidation-sensitive methionine residues can complicate chemical synthesis and handling of the peptide to be designed, we chose an M48L variant as a starting point for our design (Figure 2, *M48L*). To install the SOD activity on the SH3 domain, we used the Match protocol of Rosetta3, which allows the transfer of a function, such as specific ligand binding, from a natural model to a selected peptide/protein scaffold (Figure 1).^47^

We only considered the Cu^2+^ binding site in hSOD1 for our design. The coordination of each of the His residues to the metal center was defined by six parameters that were taken from the natural model: one distance, two angles and three dihedral angles (see Figures 1A and 1B). The Match application mutated a set of allowed positions to His in the defined orientation on the SH3 domain scaffold. If all three mutations were accommodated on the peptide backbone, an output was created (Figure 1D). The best output model displayed the potential metal binding site at the original binding groove of the natural peptide ligand of the SH3 domain (see Figure 2, *match*). The matches were then optimized with Rosetta Design giving *preSO1* as sequence with lowest energy score (Figure 2, *preSO1*). Aspartic acid residues in critical positions for aspartimide formation were replaced by glutamic acid or asparagine and V16 was varied to glutamine to better resemble the native loop in the wildtype. The resulting sequence was dubbed *SO1* (Figure 2). SO1 was synthesized by automated microwave-assisted solid-phase synthesis using a protocol by Palasek et al. with reduced heat and extended reaction time in the coupling step to reduce side product formation (for details see Figure S2).^48^

### SO1 Is a Stably Folded Protein

**Figure 3:**
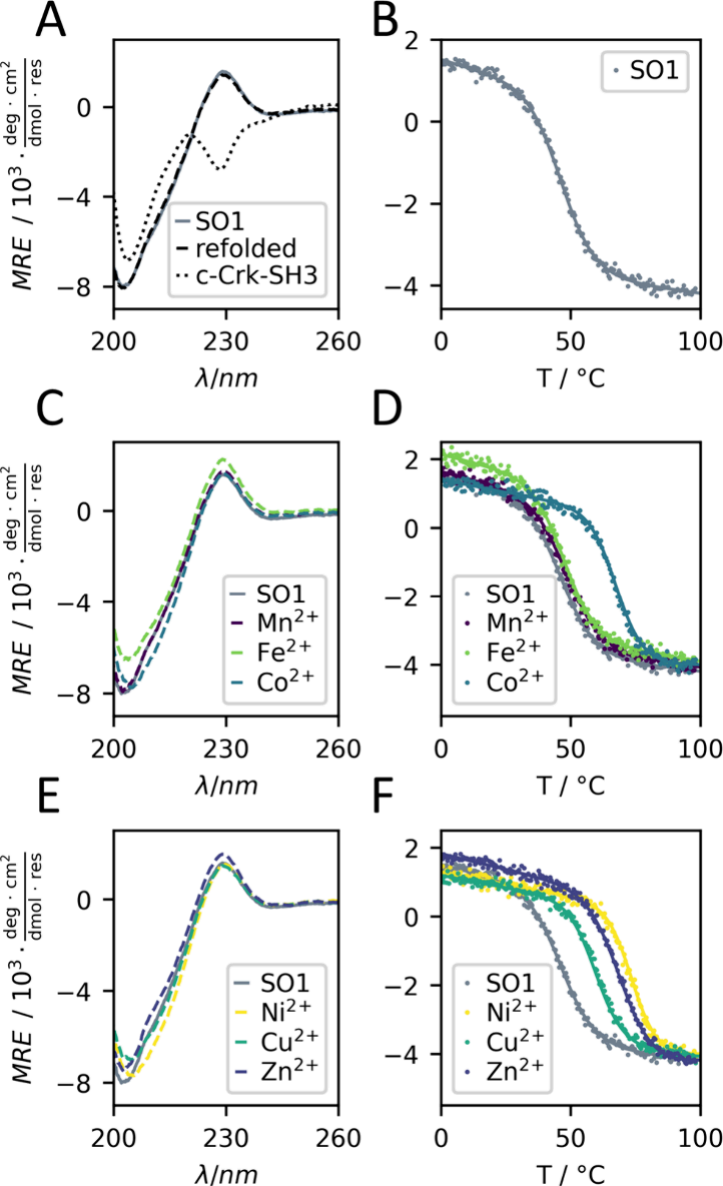
CD analysis of SO1 in the absence and presence of divalent metal ions. (A) CD spectrum at 20 °C and (B) thermal denaturation profile of SO1-apo; (C) CD spectra and (D) thermal denaturation profiles of SO1 in the presence of Mn^2+^, Fe^2+^, and Co^2+^; (E) CD spectra and (F) thermal denaturation profiles of SO1 in the presence of Ni^2+^, Cu^2+^, and Zn^2+^(conditions: 15 μM SO1 (dimeric protein, *vide infra*), 30 μM metal salt (if present), 10 mM MOPS, 150 mM NaCl, pH 7.2; thermal denaturation profiles were recorded at 228 nm).

The structure and thermal stability of SO1 was analyzed by circular dichroism (CD) spectroscopy (Figure 3). Interestingly, the CD spectrum of SO1 differed significantly from that of the wildtype c-Crk-SH3. In the CD spectral region around 228 nm, the spectrum of c-Crk-SH3 shows a characteristic minimum, whereas the spectrum of SO1 displays a maximum at that wavelength. This difference indicates major changes in the three-dimensional structure of engineered SO1 compared to the wildtype, as this signal results from exciton coupling of the aromatic residues in the hydrophobic core. However, thermal denaturation experiments revealed good thermal stability of the folded peptide, showing a reversible folding-to-unfolding with a melting temperature (*T*_m_) of 47 °C.

Despite the structural deviations from c-Crk-SH3, we recorded CD spectra and thermal denaturation curves of SO1 in the presence of first-row transition metal ions – Mn^2+^, Fe^2+^, Co^2+^, Ni^2+^, Cu^2+^, Zn^2+^ – to investigate the binding to divalent metal ions. While only minor structural changes were observed in the CD spectrum upon addition of metal ions, this was not the case for the thermal stability. An increase in the melting temperature of SO1 was observed in the presence of each of the six divalent metal ions investigated. Mn^2+^ and Fe^2+^ only increased the thermal stability slightly by about 1–2 K, whereas addition of Co^2+^, Ni^2+^, Cu^2+^ and Zn^2+^ resulted in considerable increases in *T*_m_ of up to 27 K in the case of Ni^2+^ (Table 1).

**Table 1:** Melting Temperatures and Binding Dissociation Constants of SO1 and SO1 Metal Complexes

			competitor		
	*T*_m_ [°C]	*K*_d_ [M]		λ [nm]^a^	*K*_d_ [M]^b^
SO1-apo	47 ± 0.5				
SO1-Mn^c^	49 ± 1.0	1.09 ± 0.01 × 10^–6^	MagFura-2^49^	324/365	8.9 × 10^–7^
SO1-Fe	48 ± 0.5				
SO1-Co^c^	68 ± 0.5	4.75 ± 0.01 × 10^–7^	MagFura-2^49^	324/365	9.2 × 10^–7^
SO1-Ni^c^	74 ± 1.0	4.62 ± 0.01 × 10^–8^	Fura-2^50^	335/365	6.9 × 10^–9^
SO1-Cu^d^	60 ± 1.0	3.20 ± 0.02 × 10^–9^/1.38 ± 0.22 × 10^–6^	TAMSMB^51^	586	4.4 × 10^–8^
SO1-Zn	70 ± 1.0	3.38 ± 0.01 × 10^–8^	MagFura-2^49^	324	3.7 × 10^–8^

a Titration was monitored at the wavelength of the absorbance maxima. The change in absorbance intensity was measured for Cu^2+^ and Zn^2+^ titration experiments. The change in the ratio of the absorbance maxima was measured for the Mn^2+^, Co^2+^, and Ni^2+^ titration experiments.

b Binding dissociation constants of the competitor to the respective first-row transition metal ion (see also Figures 3 and 4).

c A one-site model has been applied to determine the *K*_d_ value (for further information see the SI).

d A two-site model has been applied to determine the *K*_d_ value (for further information see the SI).

### SO1 Binds to First-Row Transition Metals

Binding of SO1 to first row transition metal ions was quantified by competitive binding experiments. Binding of Fe^2+^ was not studied due to its oxygen sensitivity. We titrated the respective metal ion to a buffered solution of SO1 and a metal-binding dye of equal concentration, monitored the change in absorbance and calculated the *K*_d_-values from the known *K*_d_-value of the dye as the average of three titration experiments using DynaFit (Figure 4).^50,52^ An overview of the competing dyes used in the respective binding study and the resulting *K*_d_-values is given in Table 1. The determined *K*_d_-values are in the range of low μM for Mn^2+^ to low nM for Cu^2+^ following the experimentally determined trend for complex stabilities of first row transition metals of the Irving-Williams series with Mn^2+^ < Co^2+^ < Ni^2+^ < Cu^2+^ > Zn^2+^.^53^ Interestingly, the *K*_d_-value of Cu^2+^ is only one order of magnitude lower than that of Zn^2+^ and two orders of magnitude lower than that of Co^2+^, which is unusual considering that Cu^2+^ complexes are especially stable due to the Jahn–Teller distortion. Also, a two-site binding model fitted the titration data from Cu^2+^ binding significantly better than a one-site binding model giving *K*_d_-values of 3.2 nM and 1.38 μM. The binding data, as well as the CD spectroscopic data, thus point to a structure of SO1 that deviates strongly from an SH3 domain scaffold of c-Crk-SH3.

**Figure 4:**
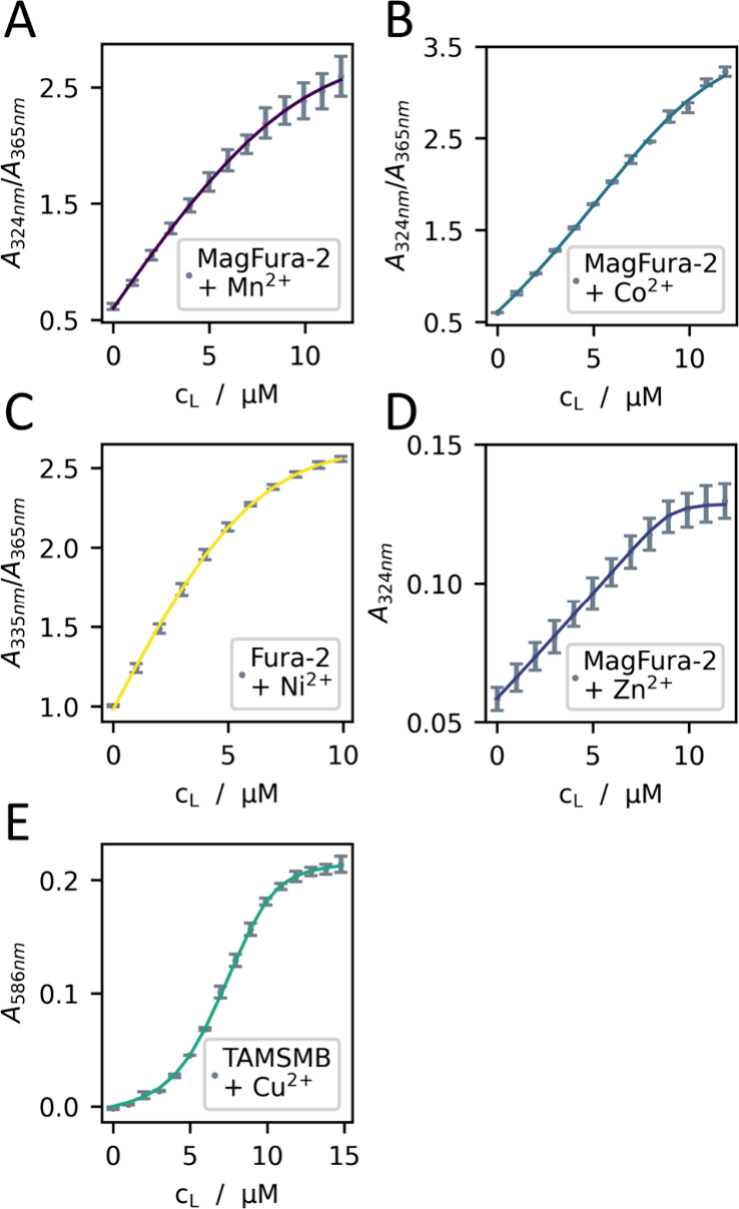
Competitive binding experiments of SO1 with first-row transition metals. (A) SO1 and Mn^2+^ in the presence of Mag-Fura-2; (B) SO1 and Co^2+^ in the presence of Mag-Fura-2; (C) SO1 and Ni^2+^ in the presence of Fura-2; (D) SO1 and Zn^2+^ in the presence of Mag-Fura-2; (E) SO1 and Cu^2+^ in the presence of TAMSMB (conditions: 2.5 μM SO1 (dimeric protein, *vide infra*), 5 μM competitor in 2 mL buffer, 2 μL of 1 mM metal salt per titration step; the final concentration of the added metal salt and the protein has been corrected for dilution, 10 mM MOPS, 150 mM NaCl, pH 7.2).

### SO1-Cu Is a Strand-Swapped Dimer of Two SH3 Domains with Two Binuclear Copper Centers

**Figure 5:**
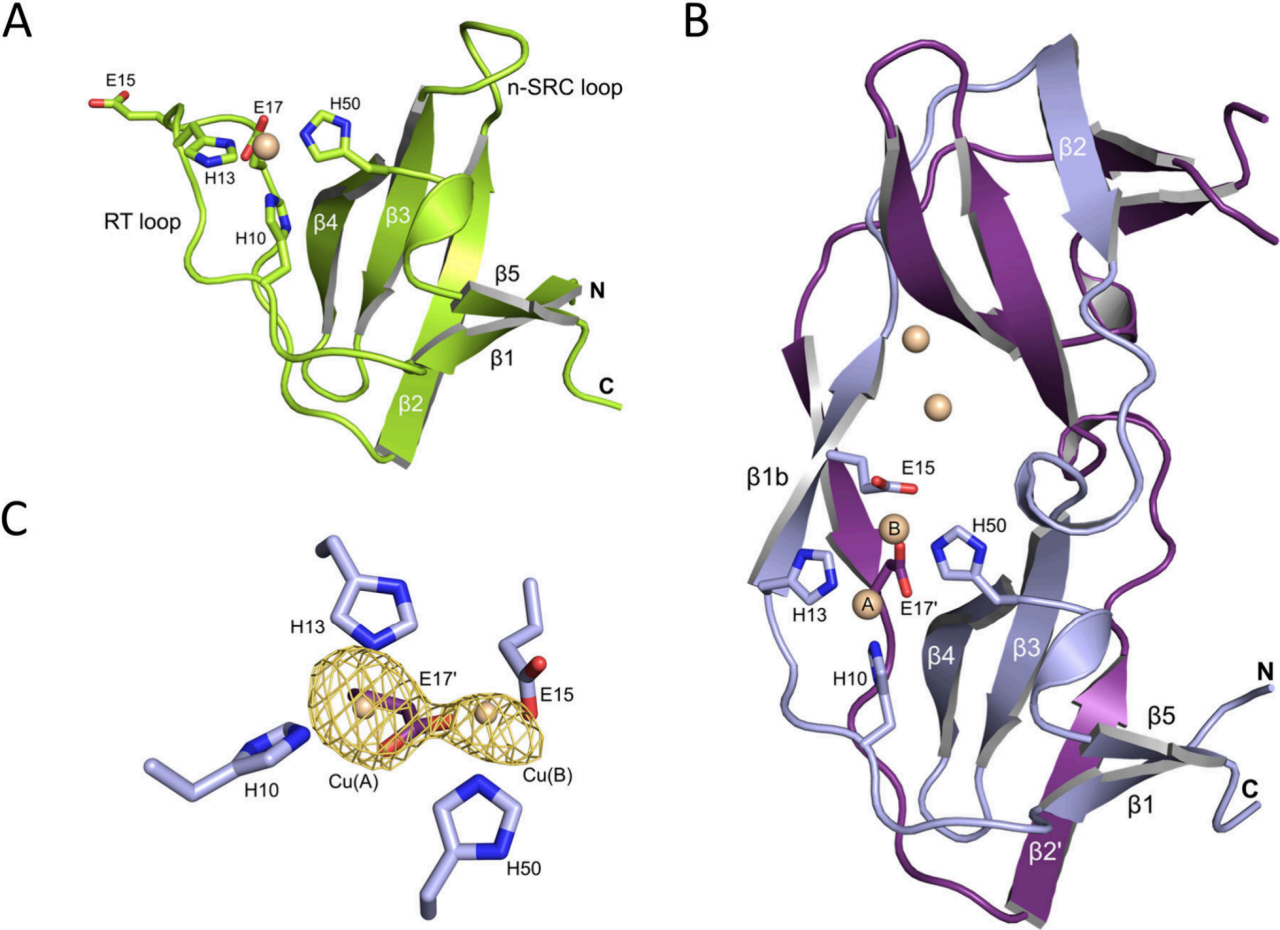
X-ray crystal structure of SO1. (A) SO1 model suggests the canonical SH3 fold. Residues H10, H13, E17, and H50 (shown as sticks) form the newly designed metal binding site. E15 is located at the tip of the RT loop. (B) X-ray crystal structure shows the strand-swapped SO1 dimer formed by chain A (lightblue) and the symmetry equivalent chain A′ (violet). Strands are labeled for one protomer and the swapped strand. The bound copper ions are represented as spheres (wheat). One of the two binuclear metal centers is shown in detail with Cu_A_ and Cu_B_ and coordinating residues H10, H13, E15, E17′, and H50 as sticks. (C) The two copper ions bound in a SO1 protomer are shown together with the anomalous density (yellow) at a contour level of 3 σ.

The ‘different from design’ nature of SO1 required us to elucidate the structure of the engineered SH3 domain-derived protein. Using X-ray protein crystallography, we determined the three-dimensional structure of SO1 with bound Cu^2+^ by molecular replacement at 2.0 Å resolution (experimental details described in the SI and Table S1). The structure is well ordered and comprises all 58 SO1 residues. Surprisingly, the structure of SO1 does not show the anticipated compact SH3-like fold of the designed model (Figure 5A). Instead, SO1 forms a homodimer in which strand β2 of the canonical SH3 fold and the adjacent loop regions are swapped with a symmetry-related molecule (Figure 5B). In detail, part of the loop between strands β1 and β2 (RT Loop residues; hinge 1) starting at residues 15, strand β2 itself (residues 23–30) and the loop between β2 and β3 (n-Src Loop residues; hinge 2) up to residue 35 are involved in the strand swapping. Dimerization creates a new antiparallel β-sheet formed by residues 13 to 17 (strand β1b) and a short 3_10_-helix before β3. There is no steric or conformational stress in the two hinge regions as there are no main chain Ramachandran or side chain conformation outliers present. Most interestingly, the dimeric structure shows two identical metal binding sites, each with two copper ions Cu_A_ and Cu_B_. Cu_A_ is coordinated by NE2 of H10 and H13. Cu_B_ is located 3.6 Å apart from Cu_A_ and is coordinated by NE2 of H50 and the carboxyl group of E15. Additionally, Cu_A_ and Cu_B_ are coordinated by the E17′ carboxyl group, which is part of the symmetry-related protein chain forming the dimer. The position of the two metal ions in the binuclear cluster were confirmed using anomalous diffraction data depicted by anomalous density in Figure 5C.

As shown in Figure S3A, strands β1 to β5 of the designed model for SO1 superpose well with strands β1, β2′ and β3 to β5 of the experimental structure. Detailed comparison of the metal binding site (Figure S3B) shows similar positions for the side chains of H10, located at the start of the RT Loop, and H50 which is at the end of β4. Due to the strand swap, H13 is more than 2 Å away from the modeled position. In the model, E15 is not involved in metal binding and located at the tip of the RT Loop. Due to the strand swap and dimerization in the experimental structure, E15 is rotated toward the copper binding site and located in the newly formed strand β1b. E17 is the fourth side chain attributed to metal binding in the SO1 model. In the experimental structure, E17 has a significantly different position and is located at the end of strand β1b. Surprisingly and coincidentally, the carboxyl group of E17′ is only about 2 Å apart from the position of modeled E17. In summary, H10 and H50 retain the modeled position, H13 is present but shifted, E15 is rotated into the binding site and the position of E17 is coincidentally taken by E17′.

Next, we searched the PDB for similar strand-swapped SH3-like structures using DALI.^54^ Two structural homologues could be found, namely PDB entries 4le9 and 7pvw.^55^ Both structures contain identical mutations in the n-Src and RT Loops of the SH3 part of tyrosine-protein kinase Src from chicken and only differ in the N-terminal residues from the purification tag. Superposition of 4le9 onto our structure resulted in a rmsd(C_α_) of 2.3 Å for 52 aligned residues which gave a sequence identity of 25% (Figure S4). Most noteworthy, structure prediction using the public AlphaFold3^56^ server with a single SO1 sequence and one copper ion as input resulted in five models that showed the compact canonical SH3 domain fold. Additionally, the resulting five predictions using AlphaFold3 with two SO1 sequences and four copper ions also showed the canonical SH3 fold. Moreover, none of the predictions using AlphaFold2 in multimer mode with two SO1 sequences did result in a 3D model showing a similar strand swap as observed in our structure (Figures S5A–S5C).^200^

In order to test whether the dimer exists in solution, we performed size exclusion chromatography to determine the differences in the elution volumes of SO1 and SO1-Cu compared to wildtype c-Crk-SH3. While there is no difference in the elution volume of SO1-apo and SO1-Cu, c-Crk-SH3 elutes later indicating a slightly smaller apparent molecular weight (Figure S6). This suggests that SO1 exists as a dimer in solution and that not only the holo form but also the apo form is dimeric. Note that we have not obtained a crystal structure of SO1-apo. We then performed electron paramagnetic resonance (EPR) spectroscopy to determine the binding mode of the SO1-Cu complex. To our surprise, SO1-Cu is EPR silent, with only traces of free Cu^2+^ detected in the solution of the protein complex (Figure S7). Finally, we also recorded the CD spectrum and the thermal denaturation curve at a four-fold excess of Cu^2+^, both showing only small but measurable differences compared to the CD data measured at a 1:2 ratio of SO1 and Cu^2+^ (Figure S8). While the CD spectrum shows a higher intensity of the exciton signal, the melting temperature has decreased by 4 K to 58 °C.

### SO1-Cu Shows Superoxide Dismutase Activity

**Figure 6:**
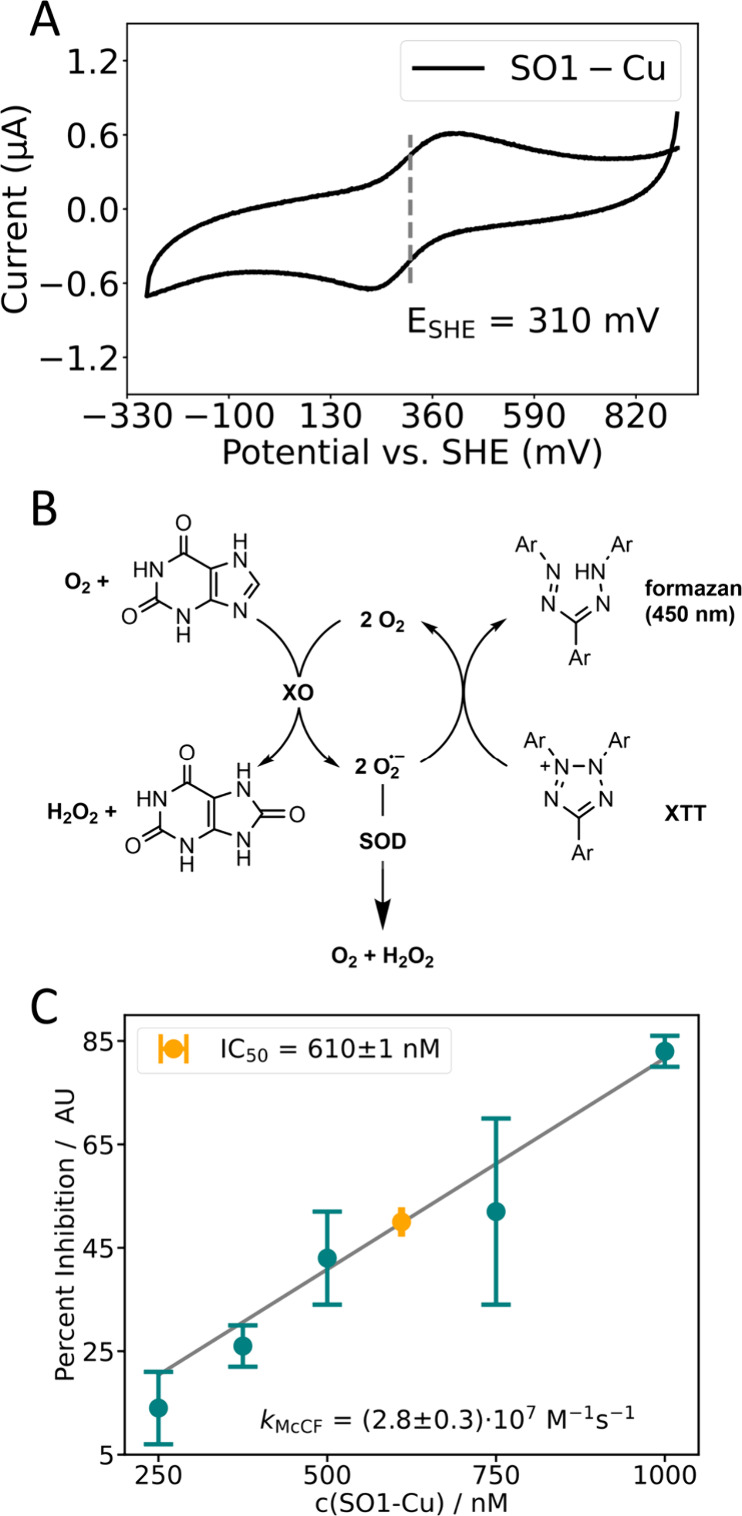
Redox activity of SO1-Cu. (A) Cyclic voltammogram SO1-Cu at pH 6.0. The reduction potential is 0.31 V against NHE. The peak separation is 122 mV which indicates semireversibility. Conditions: 10 mM MES, 150 mM NaCl, pH 6.0. SO1-Cu was drop-casted on a clean pyrolytic graphite edge and the peptide film stabilized by a layer of Nafion. Scanning speed 10 mM·s^–1^. (B) Reaction scheme of the McCord-Fridovic assay. (C) Linearized activity rate plot from competitive activity experiments with XTT at different concentrations of SO1 (IC_50_ marked in orange, Figure S10). Conditions: 200 μM XTT, 100 μM xanthine, SO1:CuSO_4_ (1:1 in all cases), 50 mM HEPES buffer, pH 7.8. Data were obtained as a mean of three measurements.

As a first step into redox activity of SO1-Cu we initially conducted cyclic voltammetry experiments to determine the reduction potential of SO1-Cu (Figure 6A). Since the diffusion coefficient of proteins is much larger than for small molecules, direct electrochemistry as used for the latter can be challenging. For this reason, we decided to employ Protein Film Voltammetry (PFV). To this end, a layer of protein was drop-casted on a clean pyrolytic graphite edge (PGE) electrode. To help with the stability of the film, an additional layer of Nafion was added to the peptide layer. It is worth noting, that we could only observe a well-defined signal at pH 6, already increasing the pH to 6.8 caused a substantial drop of signal intensity and at pH 7.8 the signal disappeared completely even at very low scan rates (1 mV · s^–1^, Figure S8A). This phenomenon has been observed in voltammetric studies with the natural enzyme.^57^ At pH 6 and 10 mV · s^–1^ scanning speed, we could detect a strong signal that we assigned to the SO1-Cu^2+^/SO1-Cu^+^ reduction pair as this was not observed for SO1-apo nor the clean electrode (Figures S9B and S9C). The process is semireversible with a peak-to-peak separation of 122 mV centered around 310 mV vs NHE. This reduction potential is in the range of Cu,Zn SODs with hSOD1 displaying a reduction potential of 320 mV vs NHE and bSOD1 of 360 mV against NHE.^28^

We then pursued our initial aim of this study and investigated SO1 for superoxide dismutase (SOD) activity. To obtain a second order rate constant of the superoxide dismutation, we used the McCord–Fridovich (McF) assay, a well-established indirect assay that uses the tetrazolium salt XTT (sodium 2,3-bis(2-methoxy-4-nitro-5-sulfophenyl)-2H-tetrazolium-5-carboxanilide), for which the second order rate constant of formazan formation by superoxide is literature-known, to determine the rate constant *k*_McF_ of superoxide conversion of an SOD mimic from its IC_50_ value.^58^ In this assay superoxide is formed *in situ* from xanthine and bovine milk Xanthine Oxidase (XO) (Figure 6B). The calculated rate constant *k*_McF_ is comparable to the second order rate constant of the catalytic reaction *k*_2_. Since the second Cu^2+^ binding site has a *K*_d_ value for Cu^2+^ in the μM range, we did not determine the activity for the binuclear SO1-Cu species, as the quantitative population of this binding site is challenging at the nanomolar concentrations used. A large excess of Cu^2+^ would be required to fully populate both binding sites at the low protein concentrations (≤1 μM). However, such a high Cu^2+^ excess would compromise the structural integrity of SO1 (see Figure S10). In order to obtain conclusive results on the SOD activity of SO1, we decided to determine the activity of the mononuclear SO1-Cu species only, using an assay based on previously described protocols in which SO1 is used in stoichiometric amounts with Cu^2+^ to ensure that no free Cu^2+^ is present in the solution.^40,58^ We determined an IC_50_ of 610 nM for SO1-Cu of the superoxide-mediated XTT conversion (Figure 6C, Figure S11) and from that calculated the *k*_McF_ (2.8 · 10^7^ M^–1^ s^–1^). Controls with SO1-apo, SO1-Co, SO1-Zn, and free Cu^2+^ showed no inhibition (Figure S10).

The determined rate constant characterizes SO1 not only as the first designed β-sheet protein model with low complexity and SOD activity, but also as the one with the highest activity to date. SO1 is only 70-fold less active than bSOD1 and 40-fold less active than SOD5. Compared to other reported SOD mimics, SO1-Cu is approximately comparable in activity to OCP1 by Vincent et al.^59^ and the Cu^2+^ complexes presented by Csire et al.,^60^ which bind Cu^2+^ with four His residues (Table 2). However, unlike these examples, SO1 has a defined three-dimensional structure. Other low-complexity protein models such as the α-helical SOD-active coiled-coils of Singh et al.^39^ and Mathieu et al.^40^ or the thioredoxin mutant of Benson et al.^42^ have one to two orders of magnitude lower activity. These protein SOD models bind the metal center with three His residues, but are α-helical. In SO1 the protein structure around the copper binding site displays a higher similarity to the natural model SOD1.

**Table 2:** Activities of Selected Superoxide Dismutases and Superoxide Dismutase Models

	sequence length	metal ion	ligand^a^	*k* [M^–1^ s^–1^]
bovine Cu,Zn-SOD1^61^	(152)_2_	Cu^2+^, Zn^2+^	protein	1.9 × 10^9^
fungal Cu-SOD5^62^	228	Cu^2+^	protein	1.1 × 10^9^
[Cu(II)(L)(H_2_O)_2_(β-GCD)]^36^		Cu^2+^	β-GCD	1.8 × 10^8^
[Cu(II)(L)(H_2_O)_2_(β-CD)]^36^		Cu^2+^	β-CD	9.9 × 10^7^
Ac–S3H4–NH_2_^60^	7	Cu^2+^	peptide	6.5 × 10^7^
SO1-Cu	**(58)**_**2**_	**Cu**^**2+**^	**miniprotein**	**(2.8 ± 0.3)** **× 10**^**7**^
OCP1^59^	8	Cu^2+^	peptide	2.4 × 10^7^
Cu(II)/(HKHGPG)_2_/Zn(II)^63^	12	Cu^2+^, Zn^2+^	peptide	8.2 × 10^6^
S2-Thioredoxin^42^	109	Fe^2+^	miniprotein	6.2 × 10^6^
GRα_3_D H_3_^40^	98	Cu^2+^	miniprotein	3.0 × 10^6^
MHB^39^	63	Mn^2+^	miniprotein	3.7 × 10^5^

a β-GCD, mono-6-deoxy-6-guanidinocycloheptaamylose cation; β-CD, β-cyclodextrin.

## Discussion

In this study, we redesigned a natural SH3 domain, the c-Crk-SH3 domain, to bind Cu^2+^ with the aim of obtaining a low complexity protein model with superoxide dismutase activity. As a natural model, we chose the Cu^2+^ binding site of hSOD1 and used Rosetta3 to engineer a similar active site into c-Crk-SH3 (Figure 1). The designed low complexity protein was found to bind first row transition metals, with Cu^2+^ being most strongly bound. However, data analysis of the competitive Cu^2+^ binding experiment revealed two Cu^2+^ binding sites with the first *K*_d_ value in the low nanomolar range and the second *K*_d_ value in the low micromolar range (Figure 4, Table 1). In addition, structural analysis by CD spectroscopy showed large structural differences between the modified SO1 and c-Crk-SH3 (Figure 3A). Both experimental observations were confirmed by X-ray crystallography and EPR spectroscopy (Figure 5, Figure S7). EPR spectroscopy revealed a silent spectrum with only weak signals corresponding to free Cu^2+^ aquo complex visible, indicating antiferromagnetically coupled copper ions. While structure prediction using AlphaFold2 and AlphaFold3 suggests that SO1 is folded as an SH3 domain scaffold, which would not allow accommodation of a binuclear Cu^2+^ center, X-ray crystallography revealed a strand-swapped SO1 dimer with a binuclear Cu^2+^ site in each protomer. Interestingly, we did not obtain a crystal structure of the apo-protein, but since there is no difference in the size between the apo- and holo-protein (see SEC, Figure S6), data strongly suggests that SO1-apo is also a strand-swapped SH3 domain dimer, which is likely to be structurally too dynamic without Cu^2+^ to form a sufficiently stable assembly required for crystallization. This is also indicated by the melting temperatures of SO1-apo and SO1-Cu, the latter being 13 K higher.

While the strand-swapped SO1 dimer appears to be an unusual structure at first, we have identified a similar strand-swapped structure of a chimeric SH3 domain that may explain the strand swap: In both sequences there are modifications in the RT Loop that affect the local loop conformation and thus promote strand swapping (Figures S4 and S12).^55,64^ Specifically, amino acid residues with a high preference for loops, such as Asn, Asp or Ser, are replaced by residues with a similar or slightly higher preference for hinges, such as Thr, Val or Gln, thereby destabilizing the RT loop to function as a hinge region. Interestingly, an *in silico* protein aggregation study of the src SH3 domain using structure-based model simulations predicted a domain-swap mechanism for the aggregation of the src SH3 domain, with the RT loop being the hinge region, as in SO1.^64^ Despite the different nature of SO1, we tested the SOD activity of the mononuclear SO1-Cu complex and found it to be one to two orders of magnitude more active than designed low complexity proteins of the same size and only 40 to 70 times less active than natural SOD enzymes. Unfortunately, we could not conclude on the activity of binuclear SO1-Cu because the *K*_d_ value of the second copper binding site is too high to allow it to be populated at the nanomolar protein concentrations at which we have conducted the activity assay. Despite the good SOD activity of mononuclear SO1-Cu, it should be kept in mind that there are low-molecular weight complexes and nanoparticles that show impressive SOD activity close to natural enzyme activity.^33^

Although the engineering of c-Crk-SH3 did not lead to the anticipated SOD miniprotein but a protein-sized strand-swapped dimer, we consider the result to be highly interesting as it has enormous potential for future protein design endeavors.^65^ In recent years, many efforts have been made to engineer strand or domain swapping to create supramolecular protein assemblies.^65−67^ Metal-binding proteins have been at the center of interest, as metal ions confer catalytic activity to the supramolecular assembly.^67^ Among these examples is a domain-swapped azurin dimer that retains the ability to transfer electrons in the dimerized state and can be considered a prototype for the development of catalytically active domain-swapped copper proteins.^68^ Interestingly, our serendipitously designed strand-swapped protein is only the second example of a strand- or domain-swapped metalloprotein with the metal-binding site in the hinge region.^26^ SO1 has an unusual binuclear metal center. This unusual structure provides valuable information to protein designers on how to combine the two design challenges of strand swapping and a functional metal center. The high-resolution X-ray crystal structure of SO1 gives us an idea of how we might engineer the metal-binding site and the stability of the hinge loop. For example, one approach could involve engineering the β-sheet formed in the hinge region to increase stability. Previous studies, particularly on domain-swapped β-sheet proteins, have shown that engineering the hinge region can lead to highly stable domain-swapped proteins.^69,70^ However, additional studies on the structure–function relationships and dynamics of strand swapping in SO1 and variants thereof are needed to fully understand and harness this newly unlocked property. In the future, it should be possible to use SO1 as a prototype for further designs of metalloproteins with binuclear metal centers.

## Data Availability

Coordinates and structure factors are deposited in the Protein Data Bank PDB under accession code 9GGO.

## Supporting Information Available

Computational and experimental procedures; Rosetta3 scripts; and supplementary figures and tables, including visualization of the hSOD1 X-ray crystal structure (PDB 3CQP), HPLC and MALDI-TOF-MS of SO1, X-ray crystallography data collection and refinement, structural comparisons of the X-ray crystal structure of SO1 with the Rosetta3 model and the strand-swapped chimeric SH3 domain (PDB 4LE9), AlphaFold2 structure predictions, size exclusion chromatography, EPR spectroscopy, additional CD data, additional CV data, reaction kinetics of the McCord–Fridovich assay, sequence alignments of strand-swapped SH3 domain variants, and the respective parent protein domains (PDF)
