# Supplementary material for: Strand-Swapped SH3 Domain Dimer with Superoxide Dismutase Activity
Source: ACS Cent Sci. 2025 Jan 10;11(1):157–66. doi: 10.1021/acscentsci.4c01347 (PMC11758493; doi:10.1021/acscentsci.4c01347)
Supplement: Supplementary file 1 — oc4c01347_si_001.pdf [file oc4c01347_si_001.pdf]

Supporting Information

Strand-Swapped SH3 Domain Dimer with Superoxide Dismutase Activity

Florian R. Häge,[a#] Merlin Schwan,[b#] Marcos Rafael Conde González,[a,c#] Jonas Huber,[a#] Stefan Germer,[a] Matilde Macrì,[a] Jürgen Kopp,[b] Irmgard Sinning,[b*] Franziska Thomas[a*]

[a]Institute of Organic Chemistry, Heidelberg University, Im Neuenheimer Feld 270, 69120 Heidelberg, Germany

[b]Biochemistry Center, Heidelberg University, Im Neuenheimer Feld 328, 69120 Heidelberg, Germany

[c]Max Planck School Matter to Life

[#]These authors have contributed equally.

*Correspondence:

irmi.sinning@bzh.uni-heidelberg.de, franziska.thomas@oci.uni-heidelberg.de

Content

1 Supplementary Figures and Tables S3
2 Procedures S12
2.1 Modelling S12
2.2 Experimental Procedures S12
3 Appendix S18
3.1 Scripts S18
3.2 Abbreviations S26
4 References S27

1 Supplementary Figures and Tables

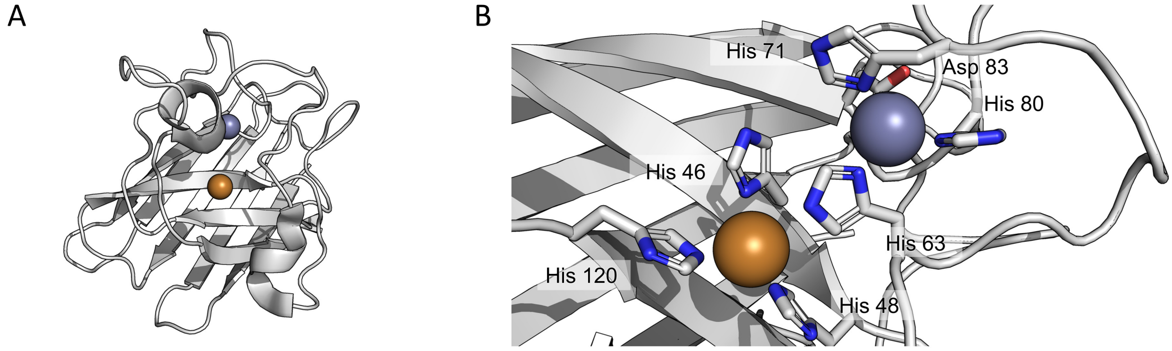

Figure S1. Ribbon presentation of hSOD1. A) hSOD1 (pdb: 3CQP) with Cu(II) (orange) and Zn(II) (grey); B) hSOD1 Cu,Zn site. Cu^{2+} and Zn^{2+} binding amino acids are shown.[1] The metal ions are coordinated tetrahedrally, with the structure-stabilizing Zn^{2+} complexed by three histidine residues (H63, H71, H80) and one aspartate residue (D83), and the catalytic Cu^{2+} complexed by four His (H46, H48, H63, H120). The two metal centers are bridged by H63.

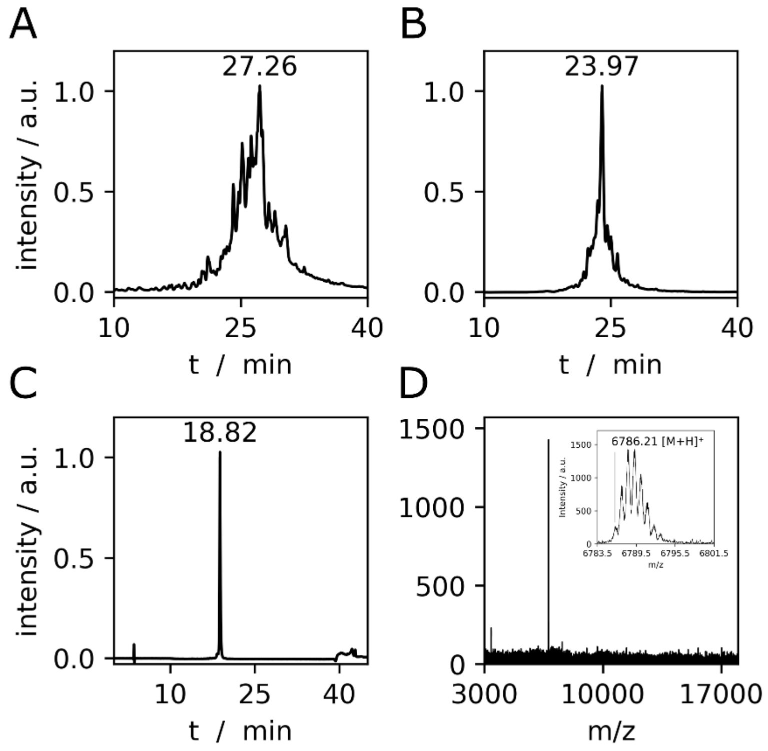

Figure S2. Characterization of SO1. A) HPLC chromatogram of synthesized SO1 with coupling steps being performed at 90 °C for 110 s (gradient: 20-40% B); B) HPLC chromatogram of synthesized SO1 with coupling steps being performed at 50 °C for 600 s (gradient: 20-50% B); C) HPLC chromatogram of pure SO1 (gradient: 20-50%B); D) MALDI-TOF-MS of pure SO1. Calculated mass for $[M+H]^+$: 6785.51.

Table S1. X-ray crystallography data collection and refinement statistics.

**SO1 (PDB 9GGO)**	
**Data Collection**	
Beamline	ESRF ID23_1
Wavelength(Å)	1.3694 Å
Resolution range(Å)	37.61 - 2.00 (2.05 - 2.00)
Space group	P 3_1 2 1
a,b,c (Å)	56.51, 56.51, 58.78
α,β,ɣ (°)	90, 90,120
Total reflections	160 381 (11181)
Unique reflections	7 674 (546)
Multiplicity	20.9 (20.5)
Completeness (%)	100 (100)
Mean I/sigma(I)	12.3 (1.5)
R-merge	0.0162 (2.814)
R-pim	0.0038 (0.653)
CC1/2	0.999 (0.833)
**Refinement**	
Resolution range(Å)	37.61 - 2.00 (2.29 - 2.00)
Reflections used in refinement	7642 (2494)
R-work	0.2034
R-free	0.2274
Number of non-hydrogen atoms	511
Macromolecules	481
Ligands	10
Solvent	20
Protein residues	58
RMS(bonds)(Å)	0.004
RMS(angles)(°)	0.650
Ramachandran favored (%)	98.2
Ramachandran allowed (%)	1.8
Ramachandran outliers (%)	0
Rotamer outliers (%)	0
Clashscore	3.98
Average B-factor	48.01
Macromolecules	47.51
Ligands	73.12
Solvent	47.50

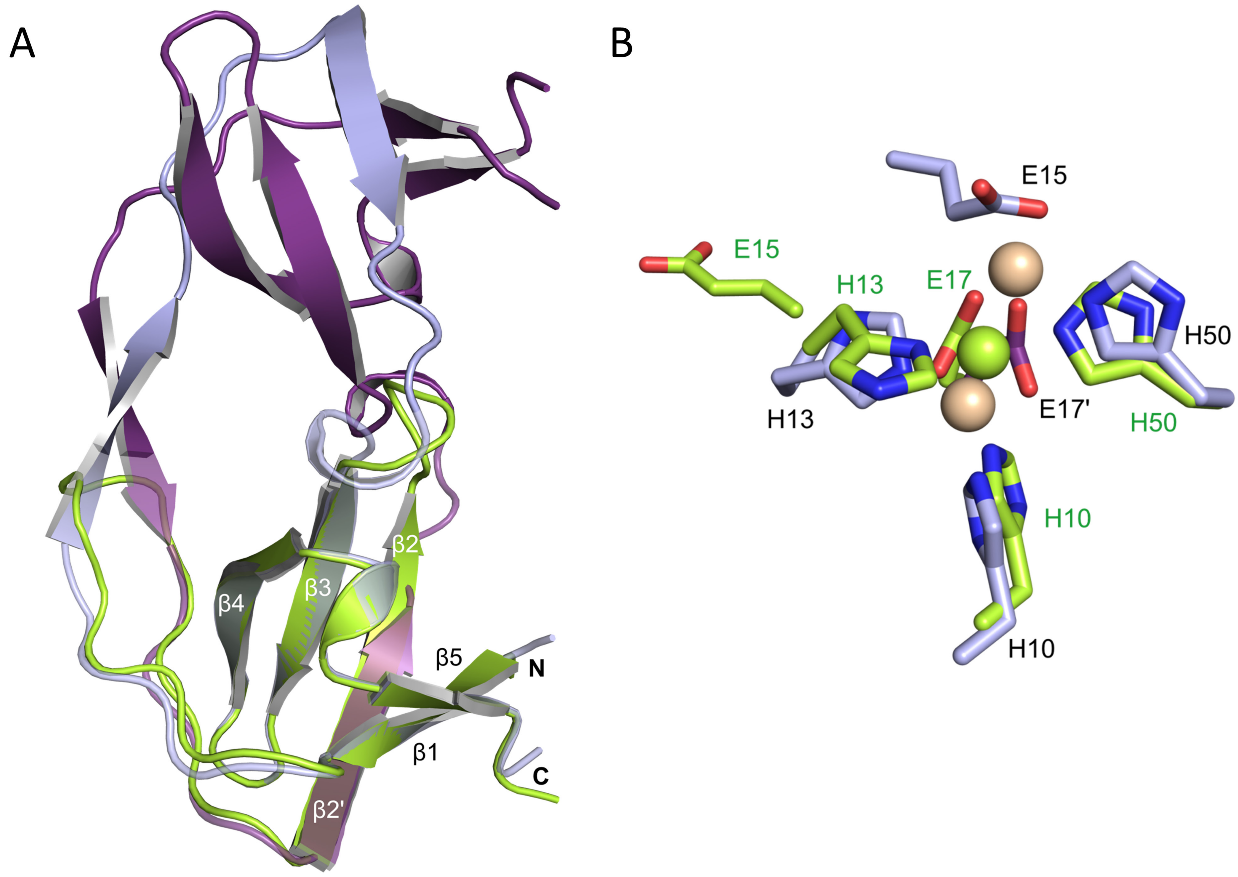

Figure S3. Comparison of Rosetta3 model and X-ray crystal structure of SO1. A) Crystal structure of the strand-swapped SO1 dimer (lightblue and violet) superposed with the designed SO1 model (green). B) Superposition of the metal binding site: only residues (lightblue, violet) coordinating the two copper ions (wheat) are shown together with the corresponding residues and copper ion of the designed SO1 model (green).

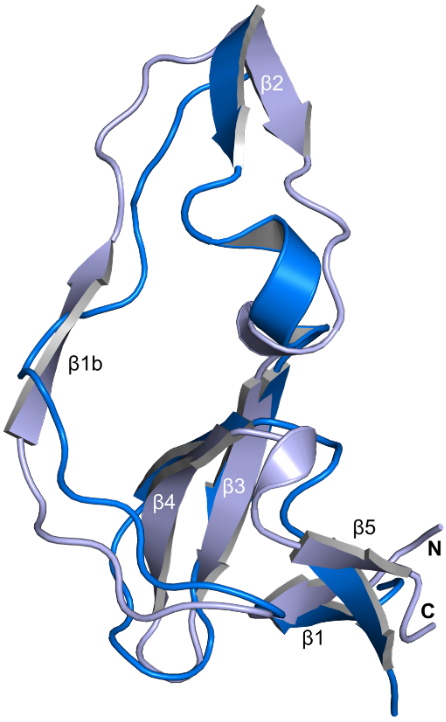

Figure S4. Structural comparison of strand-swapped SH3 domains. A superposition of a SO1 protomer (lightblue) with a protomer of PDB entry 4LE9 (darkblue).[2]

Prediction of SO1 apo state with AlphaFold

For the prediction of the apo state, we used the most recent AlphaFold2 (AF2) version (v 2.3.2) on the bwFORcluster Helix. In standard mode with a single SO1 sequence comprising the 58 residues as input, all 5 resulting predictions showed the canonical monomeric SH3-fold (Figure S5A). In multimer mode with two SO1 sequences as input, the number of models was set to five and the number of predictions per model was also set to five. Each of the five predictions per model was done with a different random seed. The resulting 25 predictions for a homo-dimer in apo state were ranked from 0 to 24 by AF2. 9 predictions (ranks 4-8, 11-13, 20) showed a domain swapped dimer, in which strand β1, RT loop, strand β2 of one protomer are binding to strands β3-5 of the other protomer (Figure S5B). This SH3 dimerization mode has been observed e.g. in Eps8 (PDB accession codes 1aoj or 1i07). 15 predictions (ranks 0-3, 9,10,14-19,21,22,24) showed dimers formed by SH3 domains retaining the canonical fold (Figure S5B). This type of dimerization has been observed e.g. in the JNK-interacting Protein 1 (JIP1; PDB accession code 7nyk). The prediction ranked as #23 is physically impossible as it contains severe steric clashes and intertwined loops. Most noteworthy, none of the predictions showed a dimer with swapped strand β2 as observed in our SO1 crystal structure. Please note, structure prediction using the public AlphaFold3[2] server run by Google Deepmind at https://alphafoldserver.com with a single SO1 sequence and one copper ion as input resulted in five models that showed the compact canonical SH3 domain fold similar to the AlphaFold2 model in Figure S5A. Additionally, the resulting five predictions using AlphaFold3 with two SO1 sequences and four copper ions also showed the canonical SH3 fold.

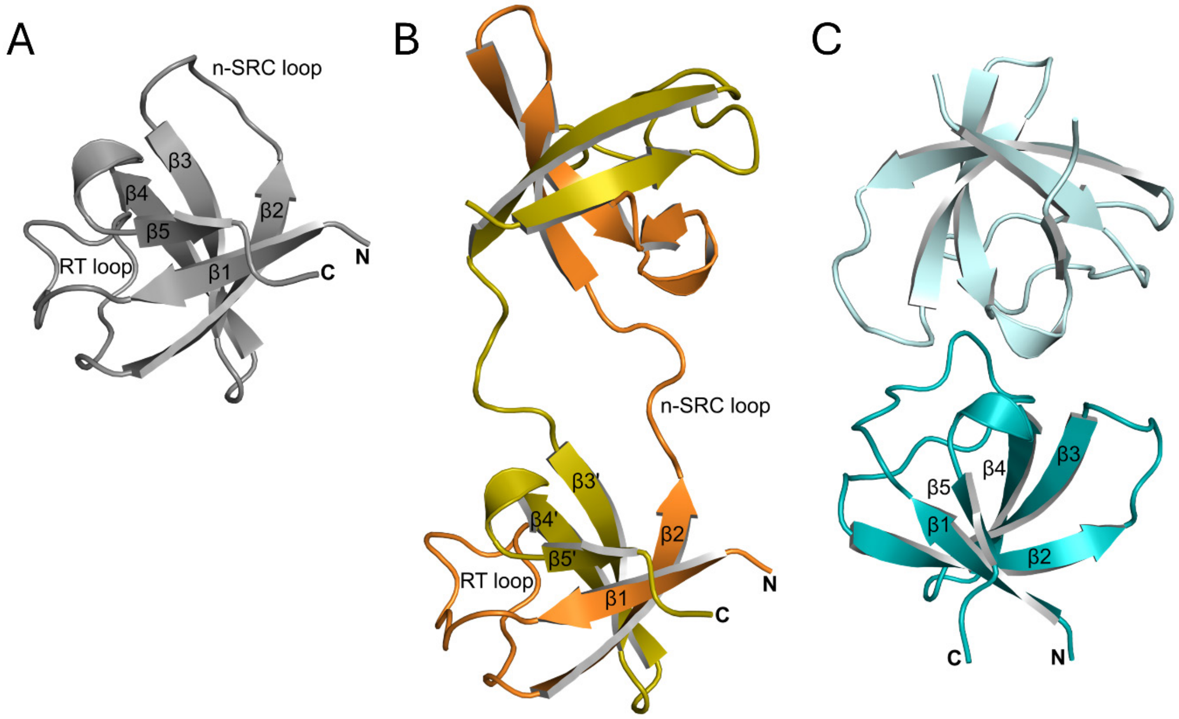

Figure S5. AlphaFold2 structure predictions on SO1. A) AlphaFold2 standard mode prediction for SO1 sequence; B) AlphaFold2 multimer mode prediction for SO1 sequence showing swap of β1, RT loop and strand β2; C) AlphaFold2 multimer mode prediction for SO1 sequence showing putative dimer with canonical SH3 fold.

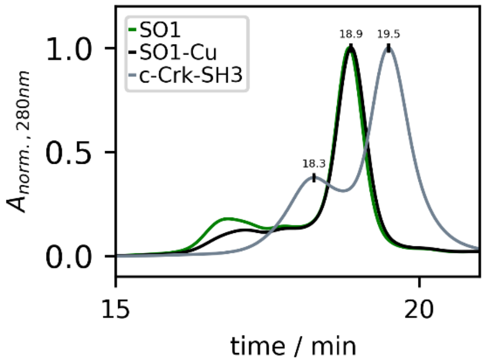

Figure S6. Size Exclusion Chromatography of SO1 and c-Crk-SH3. Size-exclusion chromatography was performed on a Superdex™ 75 increase 10/300 GL from Cytiva: Conditions: 50 mM MOPS, 150 mM NaCl, pH 7.2, flow rate: 0.8 mL · min^{-1}, peptide concentration: 50 µM (SO1 and SO1-Cu) or 10 µM (c-Crk-SH3). *The peak at 18.3 min in the SEC of c-Crk SH3 results from unspecific aggregation.

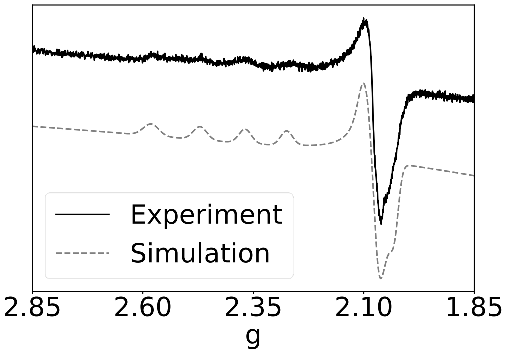

Figure S7. EPR spectroscopy of SO1. X-band EPR spectrum of SO1 depicts only free Cu^{2+}, which is confirmed by the simulated spectrum. Simulated spin Hamiltonian parameters: g_x = 2.05; g_y = 2.09; g_z = 2.42; A_z = 397 Mhz. Conditions: peptide concentration: 167 µM (50 nmol in 150 µL) with 320 µM Cu^{2+}, 10 mM MOPS, 150 mM NaCl, pH 7.4.

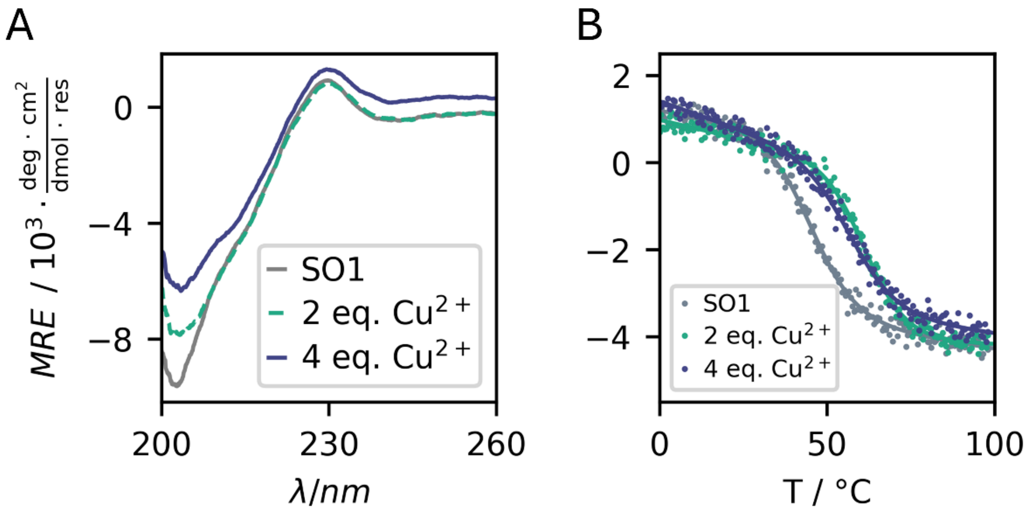

Figure S8. CD analysis of SO1 in the absence and presence of divalent 2 and 4 equivalents of Cu^{2+}. A) CD spectra at 20 °C of apo-SO1 (gray), SO1 in the presence of 2 (teal) and 4 (blue) equivalents of Cu^{2+}; B) thermal denaturation profiles of apo-SO1 and SO1 in the presence of 2 (teal) and 4 (blue) equivalents of Cu^{2+}. The Tm value of SO1 in the presence of 4 equivalents of Cu^{2+} is 58 ± 1 °C. Conditions: 15 µM SO1, 30 µM (2 equivalents) or 60 µM (4 equivalents) Cu^{2+} (if present), 10 mM MOPS, 150 mM NaCl, pH 7.2; thermal denaturation profiles were recorded at 228 nm.

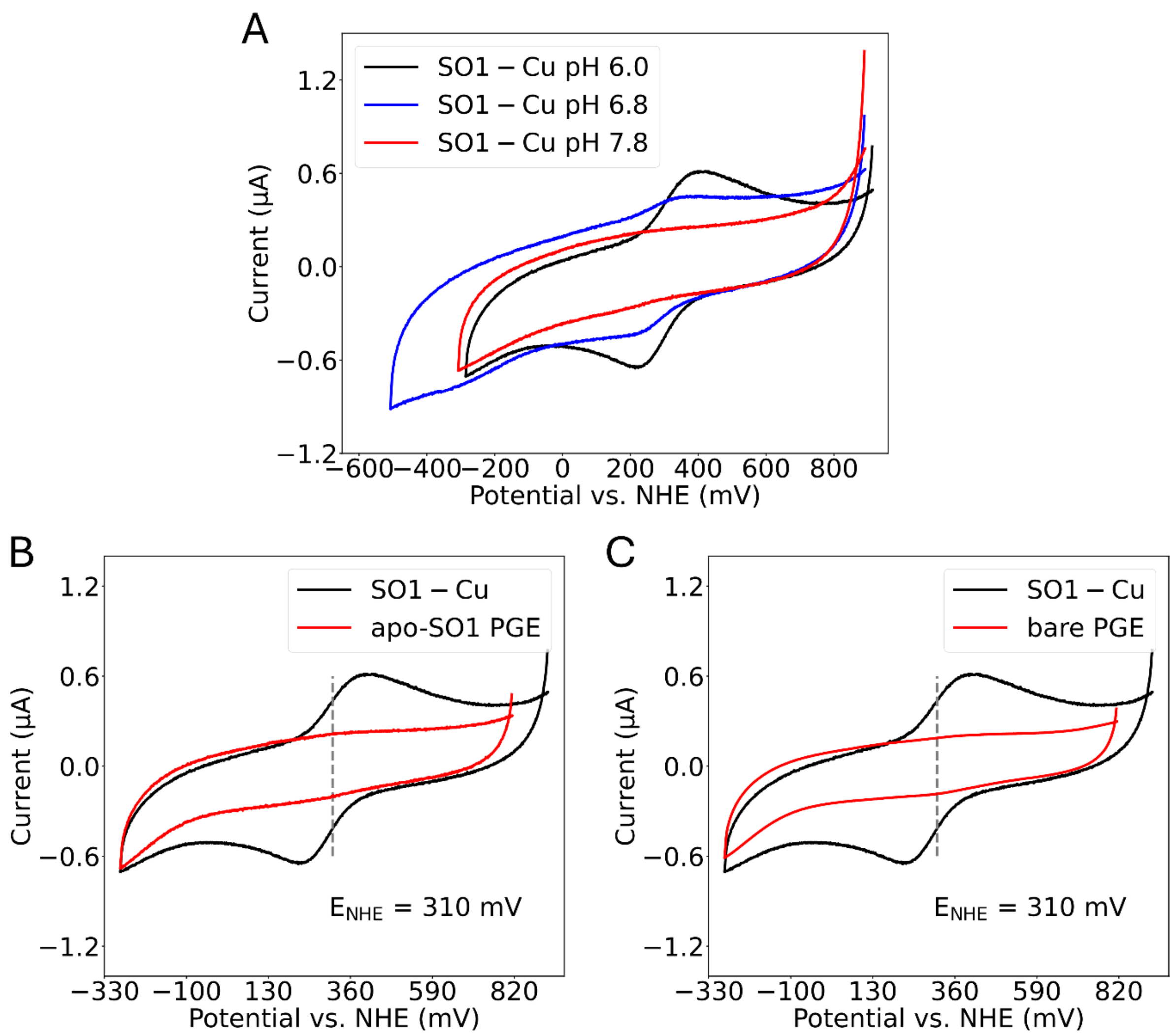

Figure S9. Cyclic voltammetry. A) Comparison of the cyclic voltammograms at pH 6.0, pH 6.8 and pH 7.8; B) comparison of the cyclic voltammograms of SO1-Cu and apo-SO1 at pH 6.0; C) comparison of the cyclic voltammograms of SO1-Cu and the clean pyrolytic graphite edge electrode at pH 6.0. Conditions: Ag|AgCl (3 M NaCl) reference electrode, platinum wire counter electrode, buffers: 5 mM MES, 200 µM KCl (pH of 6.0); 10 mM MOPS, 200 mM KCl (pH 7.4); 10 mM HEPES, 200 mM KCl (pH 7.8)

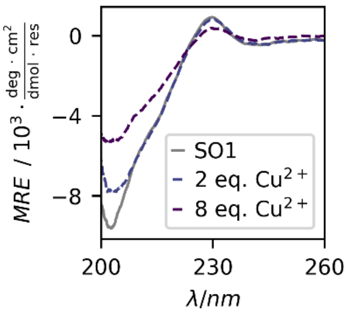

Figure S10. CD spectra of SO1 in the presence of Cu^{2+} in stoichiometric amounts and in excess. The structural integrity of SO1 is compromised, which is evident from the decrease in CD signal at fourfold excess. (Conditions: 15 µM SO1, 10 mM MOPS, 150 mM NaCl, pH 7.2. Please note: 2 eq. Cu^{2+} refers to stoichiometric amounts, since the stoichiometry of protein and Cu^{2+} in SO1-Cu is 1:2.)

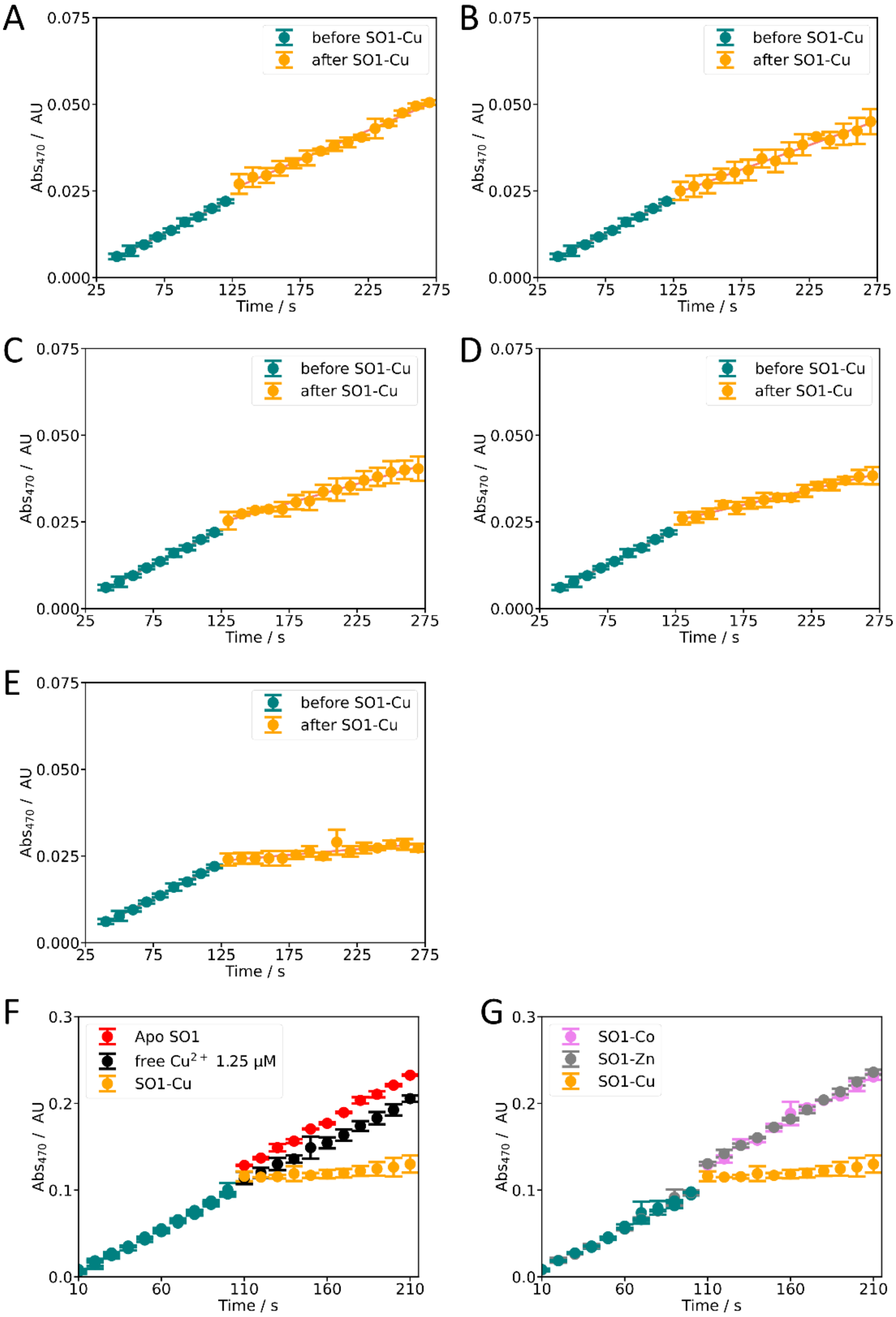

Figure S11. Reaction kinetics of the McCord-Fridovich assay on SO1-Cu. A) 250 nM SO1-Cu; B) 375 nM SO1-Cu; C) 500 nM SO1-Cu; D) 750 nM SO1-Cu; E) 1000 nM SO1-Cu. The non-inhibited reaction is shown in teal, the degree of inhibition upon the addition of SO1-Cu is shown in yellow. F) Comparison of the inhibition of the enzymatic reaction at 750 nM SO1-Cu, 1.25 μM apo-SO1 and 1.25 μM Cu^{2+} (Since for 1 μM of SO1-Cu complex close to 100% inhibition was observed, it was decided to perform the control experiment at a higher concentration of free Cu^{2+}, namely 1.25 μM, which would allow us to observe even small levels of inhibition if this was the case. Moreover, by choosing this high concentration we would also ensure that any low inhibition is not a consequence of a correspondingly low concentration of active catalytic species but reflects a property of the system.); G) comparison of the inhibition of the enzymatic reaction at 750 nM SO1-Cu, 1.5 μM SO1-Co, 1.5 μM SO1-Zn. The high concentrations of the SO1-Co and SO1-Zn were applied to detect even low activities in the assay. Color code: SO1-Cu (yellow), apo-SO1 (red), free Cu^{2+} (black), SO1-Co (pink). SO1-Zn (gray).

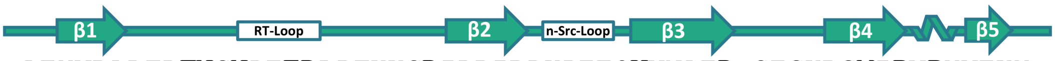

wt AEYVRALFD**FN**G**N**DE**ED**LPFKKG**D**ILRIRDKPEEQ**W**WNAE**D**-SEGKRG**M**I**P**V**P**YVEKY

SO1 AEYVRALFD**HT**G**H**DE**QE**LPFKKG**E**ILRIRDKPEEQ**L**WNAE**N**-SEGKRG**LIH**V**V**LVEKYG

c-Src-SH3 GVTTFVALYDY**ESRT**ETDLSFKKGERLQIV**NNTE**GDWWLAHSLTTG**R**TGYIPSNYVAPSD

chimeric SRC 2X GVTTFVALYDY**VASG**ETDLSFKKGERLQIV**GYNH**GDWWLAHSLTTG**Q**TGYIPSNYVAPSD

Figure S12. Sequence comparison of SH3 domains and the strand-swapped variants. Both strand-swapped dimers share a variation in the RT loop compared to their wildtype counterparts. For details on the chimeric SRC 2x we refer to Camara-Artigas et al.[3] Mutations introduced in SO1 and chimeric SRC 2X, respectively, are highlighted in bold green.

2 Procedures

2.1 Modelling

Rosetta3 Match application. The peptide models were created using Rosetta3 on an Ubuntu 20.04 LTS Windows Subsystem for Linux (WSL) on Windows 10. The match application was called with a BASH script (Script S1) that used a PDB file of the peptide scaffold (PDB code 1CKB), a CST file (S2) that defined the desired active site based on the natural model SOD1 (PDB code 3cqp, chain D) and a POS file (S3) that defined which positions may be mutated.[4]

Rosetta3 Design application. The Rosetta3 Design application was executed ten times on each Match output. We used a BASH script (S4) to call the Design application and the CST file (S2) from Match.[5]

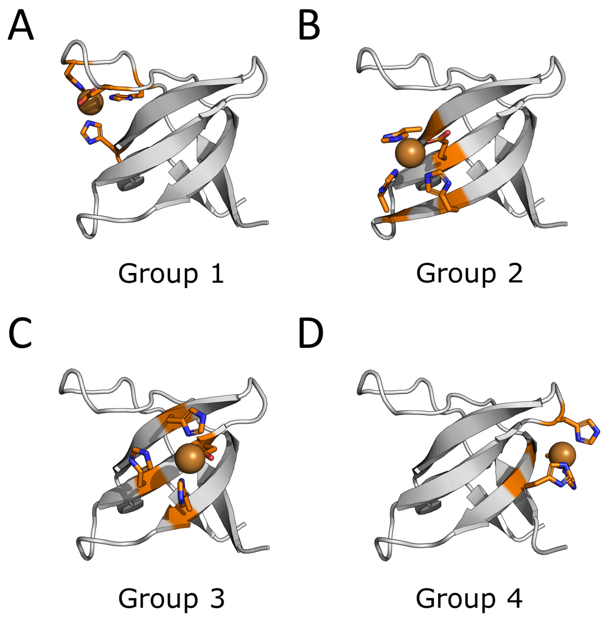

Figure S13. Rosetta Designs grouped by active site position. A) Group 1 with H10, H13, E17, H50, B) Group 2 with H30, H32, E38, H48, C) Group 3 with H29, H38, D40, H46 and (D) Group 4 with H25, H27, H42.

Rosetta3 Relax application. The Rosetta3 Relax application was executed to mutate specific positions of the sequence. We used a BASH script (S5) to call the Relax application and a RES file to define which positions were to be mutated (S6).[6]

Rosetta3 Score application. The Rosetta3 Score application was executed on the final designs to filter for aspartimide promoting sequences. We used a BASH script (S7) to call the Score application with a WEIGHTS file (S8) that penalizes aspartimide promoting sequences.[7]

2.2 Experimental Procedures

Reagents and solvents. Standard Fmoc-protected amino acids and *N,N*-diisopropylcarbodiimide (DIC) were acquired from *Iris Biotech GmbH* (Marktredwitz, Germany). Oxyma®, *N,N*-diisopropylethylamine (DIPEA), piperidine, H-Gly-HMPB-ChemMatrix® resin (production discontinued), acetonitrile (MeCN, HPLC grade), trifluoroacetic acid (TFA, HPLC grade) and triisopropylsilane (TIPS) were acquired from *Merck KGaA* (Darmstadt, Germany). DMF

(peptide synthesis grade) was purchased from *Fisher Scientific* (Loughborough, United Kingdom). All other solvents and reagents were at least *pro analysis* grade quality and were acquired from *Carbolution Chemicals GmbH* (St. Ingebert, Germany), *Carl Roth GmbH + Co. KG* (Karlsruhe, Germany), *Fisher Scientific* (Loughborough, United Kingdom), *Grüssing GmbH* (Filsum, Germany), *Honeywell* (Seelze, Germany), *Merck KGaA* (Darmstadt, Germany), *neoFroxx GmbH* (Einhausen, Germany), *SERVA Electrophoresis GmbH* (Heidelberg, Germany), *Th. Geyer GmbH & Co. KG* (Renningen, Germany) and *VWR International* (Fontenay-sous-Bois, France). Reagents and solvents were used as received. Water was purified with a *Sartorius arium® mini* lab water system and degassed by flushing with N_2-gas for 10 min.

Software for data analysis and visualization. Data were analyzed with *Python 3.10*, *DynaFit 4.11.043* and *Microsoft Excel Version 16.0* if not stated otherwise. Peptide structures were visualized with *PyMOL 2.4.0*. Chemical structures were drawn with *ChemDraw 20.0*.

Solid-phase peptide synthesis. SO1 was synthesized on a microwave-assisted peptide synthesizer (*CEM Liberty Blue*) at 0.05 mmol scale on a preloaded H-Gly-HMPB ChemMatrix® resin. A resin loading density of 0.5 mmol/g was assumed. The resin was allowed to swell in DMF for at least 10 min before the synthesis. Solutions of Fmoc-protected amino acids in DMF (0.2 M), DIC in DMF (0.5 M) as activator, and Oxyma® in DMF (1.0 M) as the activator base were used. All amino acids were coupled at 50 °C for 10 min. Single couplings were performed until the 24th amino acid, then double couplings were performed. Arginine and glycine were always double coupled. Fmoc deprotection was performed with piperidine (20 % v/v) and formic acid (5 % v/v) in DMF. After the synthesis, the peptide was transferred into a 20 mL syringe reactor with a PE frit and washed with DMF (3 x 5 mL) and DCM (10 x 5 mL) and dried under reduced pressure.
We cleaved the peptide from the resin and removed the remaining side chain protecting groups in a mixture of TFA (8.5 mL), TIPS (1 mL) and water (0.5 mL) for 3 h. The solution was collected and the resin was washed with TFA (2 x 5 mL). We removed TFA under a stream of nitrogen and precipitated the peptide from ice-cold Et_2O (25 mL). We isolated the peptide by centrifugation and washed the pellet with Et_2O (2 x 25 mL). We dissolved the peptide in a solution of acetonitrile (20 %) and TFA (0.1%) in water, froze it in liquid nitrogen and lyophilized it (*Christ Alpha 2-4-LDplus* connected to a *VACUUBRAND RZ 6* pump).

High-performance liquid chromatography (HPLC). The peptide was purified by reversed-phase HPLC on a *VDS optilab VDSpher® PUR 100 C18-SE* (250 mm x 10 mm, 100 Å, 5 µm) column at 50 °C at a flow rate of 3 mLmin^{-1} flow. A *Jasco* chromatography system with a *PU-4180* pump, a *CO-4060* column thermostat and a *UV-4070* detector was used. Analytical HPLC was performed using a *Hitachi Primaide* chromatography system containing a *1110 Pump*, a *1210 auto sampler*, *1310 column oven* and a *1430 diode array detector*. We used a *VDS optilab VDSpher® PUR 100 C18-SE* (250 mm x 4.6 mm, 100 Å, 5 µm) column at 50 °C and a flow rate of 1 mL min^{-1}. Preparative chromatograms were monitored at 220 nm and 280 nm. Analytical chromatograms were monitored from 190 to 400 nm. The mobile phase consisted of TFA (0.1%) in water (Buffer A) and TFA (0.1%) in acetonitrile (Buffer B). SO1 was dissolved in solutions of Buffer B (20 %) in Buffer A and filtered with H-PTFE syringe filters (0.2 µm pore size) from *Macherey-Nagel*. The solutions were purified in 30 min gradients of 20 – 50 % buffer B in buffer A. Fractions containing pure peptide were identified by analytical HPLC (20-50% B in 30 min) and MALDI-TOF MS, pooled and lyophilized.

Concentration determination by UV/VIS spectroscopy. SO1 was dissolved in water and the concentration was determined by measuring the absorbance at 280 nm (ε_{280} = 8480 $M^{-1}cm^{-1}$). The extinction coefficient was calculated from the sum of the extinction coefficients of Trp (5500 $M^{-1}cm^{-1}$) and Tyr (1490 $M^{-1}cm^{-1}$) residues.[8] UV/VIS spectroscopy was performed on a nanophotometer *IMPLEN NP80* at room temperature (22 to 25 °C). The sample (200 µL) was transferred into a cuvette (10 mm). Each sample was measured five times and the mean value was used for concentration calculation. The SO1 concentration was determined from the absorbance using the Beer-Lambert law.

Mass spectrometry. Matrix-assisted laser desorption ionisation time-of-flight mass spectrometry (MALDI-TOF MS) was performed on a *Bruker Autoflex Speed*. 1 µl of sinapinic acid (20 mg/mL in 3:7 MeCN/water + 0.1% TFA) was mixed with 1 µl of the sample, pipetted onto the target plate and air dried. The mass spectra were processed with *mMass*.[9]

Preparation of the SO1-Cu complex. SO1 was dissolved in purified water. After resting for five minutes the respective fivefold concentrated buffer was added. The sample was spun down for 30 seconds and rested for additional five minutes. 0.9 eq. of $CuSO_4$ in 3 µL purified water were placed on the wall of the tube and added to the solution by vortexing to prevent precipitation of the peptide. For copper concentrations above 0.9 eq, an additional 3 µL of $CuSO_4$ solution was added to provide the required copper equivalents.

CD spectroscopy. CD-measurements were performed on a *Jasco J-1700* CD spectrometer, equipped with a *Jasco PTC-510* Peltier thermostat using quartz cuvettes from *Starna*. The measuring chamber was flushed constantly with nitrogen. Settings: data pitch: 0.1 nm; scanning speed: 100 nm per min; sensitivity: low; response: 2 s; bandwidth: 1 nm. The peptide was prepared at a 15 µM concentration in pH 7.2 buffer containing 10 mM MOPS and 150 mM NaCl. CD spectra were recorded from 190 to 260 nm in 1 mm cuvettes at 20 °C. After placing the cuvette into the holder, the sample was left to equilibrate for 5 min prior to measurement. Ten spectra were recorded and accumulated. The measured ellipticity Θ (in mdeg) was converted into mean residue ellipticity MRE (in $deg \cdot cm^2 \cdot dmol^{-1} \cdot res^{-1}$) using Eq. 1 where Θ^{blank} is the blank signal, c is the peptide concentration in $mol \cdot L^{-1}$, l is the pathlength in mm and n is the number of backbone peptide bonds.[10]

$$MRE = \frac{\theta - \theta^{blank}}{c \cdot l \cdot n} \quad \textit{Eq. 1}$$

Thermal denaturation curves were recorded at 228 nm from 0 to 98 °C in 1 mm cuvettes with a temperature gradient of 1 $°C \cdot min^{-1}$. Data points were collected every 0.5 °C and fitted to a two-state model (Eq. 2) with molar enthalpy ΔH, universal gas constant R, melting temperature T_m (in K), temperature T (in K), slope of the lower plateau m, slope of the upper plateau n, abscissa of the lower plateau u and abscissa of the upper plateau v.[11]

$$f(T) = (m\,T + u) + \frac{(n\,T + v) - (m\,T + u)}{1 + \exp\left(\frac{\Delta H}{R\,T} - \frac{\Delta H}{R\,T_m}\right)} \quad \textit{Eq. 2}$$

Competitive Dye Binding Assays by UV/VIS spectroscopy. Solutions of 2.5 µM SO1 and competitor in MOPS-buffered saline (10 mM MOPS, 150 mM NaCl, pH 7.2) were prepared. As competitors TAMSMB (λ_1 = 586 nm) to determine the Cu^{2+}binding affinity, Fura-2 (λ_1 = 335 nm, λ_2 = 365 nm) to determine the Ni^{2+}binding affinity and Mag-Fura-2 (λ_1 = 324 nm, λ_2 = 365 nm) to determine the Mn^{2+}, Co^{2+} and Zn^{2+} binding affinities were used. UV/VIS spectroscopy was performed on an *IMPLEN NP80* nanophotometer at room temperature (22 to 25 °C). The sample (2.0 mL) was prepared in a cuvette (10 mm) with a magnetic stirrer. After stirring for 5 minutes the absorbance was measured at the wavelengths λ_1 and λ_2 of the respective dye for each addition (2 µL) of 1.0 mM metal ligand solution until saturation was reached. The final concentration for each titration step was corrected for dilution (0.999 µM, 1.996 µM, 2.99 µM, 3.98 µM, 4.975 µM, 5.964 µM, 6.95 µM, 7.937 µM, 8.92 µM, 9.9 µM, 10.88 µM, 11.858 µM, 12.833 µM, 13.807 µM, 14.778 µM). The data was analyzed using DynaFit applying either a one-site model (Mn^{2+}, Co^{2+}, Ni^{2+}, Zn^{2+}; **Script S9**) or a two-site model (Cu^{2+}; **Script S10**).[12] The binding dissociation constant K_d was determined as the mean of three titrations.

X-ray crystallography - Crystallization. Synthesized SO1 was dissolved in water and adjusted to the buffer condition of 10 mM MOPS pH 7.2, 100mM NaCl by addition of a 10x concentrated stock solution. $CuCl_2$ was added in a 1.5-fold molar excess. The sample was concentrated to 5 mg/mL with an *Amicon Ultra* concentrator (3 kDa cutoff) and crystallized at 291 K using sitting drop vapor diffusion technique. The reservoir consisted of 1.26 M $(NH_4)_2SO_4$, 0.1 M CHES pH 9.5 and 0.2 M NaCl. Crystallization drops contained 300 nL reservoir solution and 300 nL concentrated protein. Crystals were visible after 14 days and were flash-frozen in liquid nitrogen using ethylene glycol as cryoprotectant.

X-ray crystallography - Data Collection, Processing, Refinement. Data were collected at ESRF Grenoble beamline ID23_1 at cryogenic conditions, integrated using XDS[13] and scaled using *AIMLESS*[14] as part of the CCP4i software package.[15] Friedel pairs were treated separately for anomalous data analysis. Phases were obtained by molecular replacement using a Colabfold[16] model of SO1 with *PHASER*[17] implemented in the *PHENIX* package.[18] The final data set had space group $P3_121$ and contained one SO1 molecule per asymmetric unit. Iterative model building and refinement was performed with *Coot*[19] and *Phenix.refine*.[20] The quality of the resulting structural models was analyzed with *MolProbity*.[21] Structure figures were prepared with *PyMOL 2.5.7* (The PyMOL Molecular Graphics System, Schrödinger, LLC.). Crystallographic data are summarized in Table S1. Coordinates and structure factors are deposited at the Protein Data Bank PDB with accession code 9GGO.

Size exclusion chromatography. Size exclusion chromatography was performed on a *Cytiva Superdex 75 Increase 10/300 GL* at 25 °C at a flow rate of 0.8 mLmin-1. A *Shimadzu* chromatography system with a *LC-40D* pump, a *CTO-40S* column oven, a *SPD-M40* detector, a *DGU-405* degassing unit and a *CMB-40* system controller was used. The runs were monitored from 200 to 400 nm. The mobile phase was degassed in an ultrasonic bath for 30 minutes and consisted of 10 mM MOPS and 150 mM KCl in purified water at pH 7.4 (Buffer C). SO1 was dissolved in Buffer C, and 1 µL of $CuSO_4$ was added successively. The sample was centrifuged for 30 minutes at 14500 RPM and 100 µL were injected onto the column. Each run was stopped after 35 minutes. The data was processed with *Python 3.8.10*.

EPR spectroscopy. EPR tubes (3 mm, quartz) were filled with 150 µL of sample. The concentration of dimer was approximately 167 µM (50 nmol in 150 µL) with 320 µM Cu^{2+} in 10 mM MOPS buffer pH 7.4. An excess peptide was employed to ensure that all Cu^{2+} was in the bound form. Additionally, the solution contained 150 mg/mL of glycerol as a glassing agent. Immediately before measurement, the samples were frozen in liquid nitrogen. Spectra were recorded at 4 K on a *Bruker ElexSys e500* equipped with an *F-70 Sumitomo cryogenics* helium cryostat, *Bruker ER4116-DM* resonator, *Oxford LLT 650 low loss* transfer tube, and an *Oxford ITC503* temperature controller.

Protein Film Voltammetry. Protein Film voltammetry (PFV was carried out by adsorbing the peptide-copper(II) solution on a previously polished pyrolytic graphite edge (PGE) electrode. Shortly, the PGE electrode was cleaned by polishing with 0.05 µm alumina for 1 min, followed by washing under a gentle stream of milliQ water for 30 s and sonication in milliQ water for 5 min. It was then washed a final time for 30 s with milliQ and dried on a stream of nitrogen. Immediately after, 5 µM of a 50 µM solution of the holo -peptide in buffer and 5 µL of a 0.5% Nafion-NS5 solution were sequentially drop-casted on its surface and mixed thoroughly. The resulting mixture was dried under a gentle stream of nitrogen and kept covered until the measurements. The measurements were carried out employing a Ag|AgCl (3 M NaCl) reference electrode and a Platinum wire as counter electrode, both from BioLogic. The buffer solution contained 5 mM MES + 200 µM KCl as supporting electrolyte with final pH of 6 and it was degassed before each experiment by bubbling nitrogen for at least 15 min. During measurement, oxygen was kept off of the solution by a blanket of nitrogen on top of the cell. Cyclic voltammetry was measured on a BASi potentiostat and analyzed in *Python 3.8.10*.

SOD activity assays. The activity assays for the determination of the k_{McF} were performed as described in Sutherland et al. in 50 mM HEPES buffer pH 7.8.[22] Shortly, xanthine oxidase (XO) from the SOD1 kit from Sigma-Aldrich (CS0009) was thawed and diluted to obtain a stock which upon dilution in the final assay mixture led to a rate of urate production of ~0.02 absorbance units per minute measured at 295 nm. Xanthine was dissolved in 1 M NaOH with sonication. XTT was dissolved in water to obtain a stock of ~14 mM that was kept at 37 °C to avoid precipitation before dilution. The final solutions of both were prepared in buffer with the following concentrations: xanthine, 1 mM and XTT, 1 mM. Stock solutions of SO1-Cu were prepared in buffer in a stoichiometric ratio of apo-SO1 and Cu^{2+}. The measurements were performed on an IMPLEN NP40 nanospectrophotometer at room temperature (~23 °C) with quartz cuvettes (Hellma). Formation of the formazan dye resulting from the reduction of XTT by superoxide was followed at 470 nm. All measurements were performed in triplicate.

For the assay, stock solutions were diluted inside a quartz cuvette (Hellma®, 1 cm) to obtain a final solution of 100 µM of xanthine and 200 µM XTT in 50 mM HEPES buffer pH 7.8. The assay was started by the addition of XO and it was allowed to progress for 2.5 min to obtain the slope of the uninhibited reaction (P1). At this point, 10 µL of a solution of SO1-Cu were added and the assay continued for another 2.5 min to obtain the slope of the reaction after addition of the SOD1 analogue (P2). To find the IC_{50}, the plot of [(P1-P2)/P2]·100 vs. the concentration of SO1-Cu was fitted to a line and the IC_{50} was found by interpolating the 50% point. From the IC_{50}, the k_{McCF} was then calculated as reported by Sutherland et al..[22]

For the control experiments with different metal ions, apo-SO1 was incubated with a solution of the metal ion in a 1:1 ratio. The final concentration of each was 1.5 µM (metal ion) and 1.5 µM (SO1). This high concentration was used to detect any level of activity, even a low one. Please note: Under these conditions, the SO1-Cu complex completely inhibits the reaction of superoxide ions with the XTT. In addition, controls were carried out with apo-SO1 and free Cu^{2+}. The final concentration of each was 1.25 µM.

3 Appendix

3.1 Scripts

Script S1. BASH Script for the Rosetta3 Match application.

```
# Call the match algorithm of Rosetta.

/PATH/Rosetta3/main/source/bin/match.static.linuxgccrelease

-lig_name CU

-geometric_constraint_file 3cqp_D.cst

-in:file:s 1ckb.pdb

-scaffold_active_site_residues sh3.pos

-extrachi_cutoff 0

-ex1

-ex2

-use_input_sc

-no_opt_H false

-enumerate_ligand_rotamers false

-dynamic_grid_refinement

-match_group SameSequenceAndDSPositionGrouper

-consolidate_matches

-output_matches_per_group 1

-out:file:output_virtual
```

Script S2. Rosetta3 CST file.

```
# CST constraint file for CuHHH of SOD1 (3CQP//D)

# Block 1: His-46, Nhis/ND1 binding to Cu

CST::BEGIN

TEMPLATE:: ATOM_MAP: 1 atom_name: CU V3 V4

TEMPLATE:: ATOM_MAP: 1 residue_3: CU

TEMPLATE:: ATOM_MAP: 2 atom_type: Nhis

TEMPLATE:: ATOM_MAP: 1 residue_3: HIS

CONSTRAINT:: distanceAB: 2.28 0.3 40.0 1 1
```

```
CONSTRAINT:: angle_A: 130.1 20.0 40.0 360.0 1

CONSTRAINT:: angle_B: 123.6 15.0 40.0 360.0 1

CONSTRAINT:: torsion_A: -123.9 30.0 40.0 360.0 1

CONSTRAINT:: torsion_AB: -16.5 30.0 40.0 360.0 1

CONSTRAINT:: torsion_B: 23.7 30.0 40.0 360.0 1

CST::END


# Block 2: His-48, Ntrp/NE2 binding to Cu

CST::BEGIN

TEMPLATE:: ATOM_MAP: 1 atom_name: CU V4 V2

TEMPLATE:: ATOM_MAP: 1 residue_3: CU

TEMPLATE:: ATOM_MAP: 2 atom_type: Ntrp

TEMPLATE:: ATOM_MAP: 1 residue_3: HIS

CONSTRAINT:: distanceAB: 2.00 0.3 40.0 1 1

CONSTRAINT:: angle_A: 115.4 20.0 40.0 360.0 1

CONSTRAINT:: angle_B: 108.4 15.0 40.0 360.0 1

CONSTRAINT:: torsion_A: -140.3 30.0 40.0 360.0 1

CONSTRAINT:: torsion_AB: -87.9 30.0 40.0 360.0 1

CONSTRAINT:: torsion_B: -166.0 30.0 40.0 360.0 1

CST::END


# Block 3: His-120, Ntrp/NE2 binding to Cu

CST::BEGIN

  TEMPLATE:: ATOM_MAP: 1 atom_name: CU V2 V3

  TEMPLATE:: ATOM_MAP: 1 residue_3: CU

  TEMPLATE:: ATOM_MAP: 2 atom_type: Ntrp

  TEMPLATE:: ATOM_MAP: 1 residue_3: HIS

  CONSTRAINT:: distanceAB: 2.03 0.3 40.0 1 1

  CONSTRAINT:: angle_A: 96.2 20.0 40.0 360.0 1

  CONSTRAINT:: angle_B: 119.9 15.0 40.0 360.0 1
```

```
  CONSTRAINT:: torsion_A: -131.1 30.0 40.0 360.0 1
  CONSTRAINT:: torsion_AB: -121.2 30.0 40.0 360.0 1
  CONSTRAINT:: torsion_B: 148.1 30.0 40.0 360.0 1
  CST::END
```

Script S3. Rosetta3 Match POS file.

```
5 7 8 9 10 11 12 13 14 15 16 17 19 21 22 23 24 25 27 29 30 31 32 33 34 35 36
38 40 41 42 43 44 45 46 47 48 50 51 52 53
```

Script S4. BASH Script for the Rosetta3 Enzyme Design application.

```
# Call the design algorithm of Rosetta.
/PATH/Rosetta3/main/source/bin/enzyme_design.static.linuxgccrelease
-cst_file 3cqp_D.cst
-s $matchoutput.pdb
-enzdes:detect_design_interface
-enzdes:cut1 6.0
-enzdes:cut2 8.0
-enzdes:cut3 10.0
-enzdes:cut4 12.0
-enzdes:cst_opt
-enzdes:bb_min
-enzdes:chi_min
-enzdes:cst_design
-enzdes:design_min_cycles 3
-enzdes:lig_packer_weight 1
-enzdes:cst_min
-packing:ex1
-packing:ex2
-packing:use_input_sc
-packing:soft_rep_design
-packing:linmen_ig 10
```

```
-packing:extrachi_cutoff 0
-packing:no_opt_H false
-out:file:o scorefile_$matchnumber.txt
```

Script S5. BASH Script for the Rosetta3 Relax application.

```
# Call the relax algorithm of Rosetta.
/PATH/Rosetta3/main/source/bin/relax.static.linuxgccrelease
-database /PATH/Rosetta3/main/database
-in:file:s PEPTIDE.pdb
-relax:respect_resfile
-packing:resfile file.resfile
-nstruct 10
-relax:constrain_relax_to_start_coords
-relax:ramp_constraints false
-ex1
-ex2
-use_input_sc
-flip_HNQ
-no_optH false
-extrachi_cutoff 0
```

Script S6.1. RES file for the Rosetta3 Relax application. Reverse redundant mutations.

```
# Resfile to control for redundant mutations.

NATAA

AUTO

EX 1 EX 2

USE_INPUT_SC

start

11   A     PIKAA TN

16   A     PIKAA VQ

36   A     PIKAA LW

52   A     PIKAA VP

53   A     PIKAA LY
```

Script S6.2. RES file for the Rosetta3 Relax application. Aspartate mutations.

```
# Resfile to mutate aspartates.

NATAA

AUTO

EX 1 EX 2

USE_INPUT_SC

start

9     A     PIKAA DN

14    A     PIKAA DN

24    A     PIKAA DNE

30    A     PIKAA DN

41    A     PIKAA N
```

Script S7. BASH Script for the Rosetta3 Score application.

```
# Call the score algorithm of Rosetta.

/PATH/Rosetta3/main/source/bin/score_jd2.static.linuxgccrelease

-in:file:l PDBlist.txt

-score::weights ref2015_ap

-ex1

-ex2

-use_input_sc

-flip
```

Script S8. WEIGHTS file for the Rosetta3 Score application.

```
# beta_nov15

#    beta energy function following parameter refitting (Frank DiMaio and Hahnbeom Park), November 2015

#

#   Two sets of reference weight are provided.

#      The first is for use in "minimization context" (e.g., RTmin, min_pack, or sidechain relax).

#       The second, and default set, is for use in "packing context" (e.g. Rotamer trials or packing)

#

#METHOD_WEIGHTS ref 1.82468 3.75479 -2.14574 -2.72453 1.21829 0.79816 -0.30065 2.30374 -0.71458 1.66147 2.15735 -1.34026 -1.94321 -1.45095 -0.59474 -0.28969 1.15175 2.64269 2.26099 0.58223

METHOD_WEIGHTS ref 1.32468 3.25479 -2.14574 -2.72453 1.21829 0.79816 -0.30065 2.30374 -0.71458 1.66147 1.65735 -1.34026 -1.64321 -1.45095 -0.09474 -0.28969 1.15175 2.64269 2.26099 0.58223

fa_atr 1

fa_rep 0.55

fa_sol 1.0

fa_intra_sol_xover4 1.0

lk_ball_wtd 1.0

fa_intra_rep 0.005

fa_elec 1.0

pro_close 1.25

hbond_sr_bb 1.0

hbond_lr_bb 1.0
```

```
hbond_bb_sc 1.0
hbond_sc 1.0
dslf_fa13 1.25
rama_prepro 0.45
omega 0.4
p_aa_pp 0.6
fa_dun 0.7
yhh_planarity 0.625
ref 1
aspartimide_penalty 1.0
INCLUDE_INTRA_RES_PROTEIN
NO_HB_ENV_DEP
```

Script S9. DynaFit script - competitive binding assay – one-site model. Absorbance differs for each titration step.

```
Competitive fluorescence displacement assay to determine the Kd for both labeled and unlabeled ligand.
;_______________________________________________________________________
[task]
   data = equilibria
   task = fit
[mechanism]
   ; P = protein
   ; L = dye
   ; M = metal
   P + M <==> PM      :    Kd*   dissoc
   L + M <==> ML      :    Kd1   dissoc
[constants]
   Kd* = 1.0 ?
   Kd1 = 0.89
[responses]
   intensive
[data]
   variable  M, L, P
   set Abs.01 | resp L = 0.0002 ?, ML = 0.041 ?
[output]
   directory ./out
```

```
   rate-file outfile
[set:Abs.01]
M,microM    L,microM    P,microM    Abs
[end]
```

Script S10. DynaFit script - competitive binding assay – two-site model. Absorbance differs for each titration step.

```
Competitive fluorescence displacement assay to determine the Kd for both
labeled and unlabeled ligand.
[task]
   data = equilibria
   task = fit
[mechanism]
   ;
   ; P = protein
   ; L = ligand
   ; M = metal
   ;
   P + M <==> PM       :    Kd1   dissoc
   PM + M <==> PMM     :    Kd2   dissoc
   L + M <==> ML       :    Kd*   dissoc
[constants]
   Kd1 = 0.005 ?
   Kd* = 0.044
   Kd2 = 0.050 ?
[responses]
   intensive
[data]
   variable  M, L, P
   set Abs.01 | resp L = 0.0002 ?, ML = 0.041 ?
   plot titration
[output]
   directory ./output_dimers
[set:Abs.01]
M,microM    L,microM    P,microM    Abs
[end]
```

3.2 Abbreviations

bSOD	bovine Superoxide Dismutase
CHES	N-cyclohexyl-2-aminoethanesulfonic acid
CD	Circular Dichroism
c-Crk-SH3	C-terminal SH3 domain of human Crk
DIC	N,N'-Diisopropylcarbodiimide
DIPEA	N,N-Diisopropylethylamine
DMF	N,N-Dimethylformamide
EDTA	Ethylenediaminetetraacetate
EPR	Electron paramagnetic resonance
Et_2O	Diethyl ether
Fmoc	Fluorenylmethyloxycarbonyl
HEPES	4-(2-hydroxyethyl)-1-piperazineethanesulfonic acid
HMPB	4-(4-Hydroxymethyl-3-methoxyphenoxy)-butyric acid
HPLC	High performance liquid chromatography
H-PTFE	Hydrophilized polytetrafluoroethylene
hSOD1	human Cu/Zn Superoxide Dismutase 1
IC_{50}	Half maximal inhibitory concentration
K_d	Binding dissociation constant
MALDI	Matrix assisted laser desorption ionisation
MeCN	Acetonitrile
MeOH	Methanol
MES	2-(N-morpholino)ethanesulfonic acid
MOPS	3-(N-Morpholino)propanesulfonic acid
MRE	Mean residue ellipticity
MS	Mass spectrometry
NaOH	sodium hydroxide
PDB	Protein Data Bank
SOD	Superoxide Dismutase
SPPS	Solid-phase peptide synthesis
TAMSMB	2-(2-Thiazolylazo)-4-methyl-5-(sulfomethylamino) benzoic acid
TFA	Trifluoroacetic acid
TIPS	Triisopropylsilane
TOF	Time-of-flight
UV/Vis	Ultraviolet–visible
XO	Xanthine Oxidase
XTT	sodium 2,3-bis-(2-methoxy-4-nitro-5-sulfophenyl)-2H-tetrazolium-5-carboxanilide

4 References

(1) Gleason, J. E.; Galaleldeen, A.; Peterson, R. L.; Taylor, A. B.; Holloway, S. P.; Waninger-Saroni, J.; Cormack, B. P.; Cabelli, D. E.; Hart, P. J.; Culotta, V. C. Candida albicans SOD5 represents the prototype of an unprecedented class of Cu-only superoxide dismutases required for pathogen defense. *Proc. Natl. Acad. Sci. U. S. A.* **2014**, *111* (16), 5866-5871. DOI: 10.1073/pnas.1400137111.

(2) Abramson, J.; Adler, J.; Dunger, J.; Evans, R.; Green, T.; Pritzel, A.; Ronneberger, O.; Willmore, L.; Ballard, A. J.; Bambrick, J.; et al. Accurate structure prediction of biomolecular interactions with AlphaFold 3. *Nature* **2024**, *630* (8016), 493-500. DOI: 10.1038/s41586-024-07487-w.

(3) Cámara-Artigas, A.; Martínez-Rodríguez, S.; Ortiz-Salmerón, E.; Martín-García, J. M. 3D domain swapping in a chimeric c-Src SH3 domain takes place through two hinge loops. *J. Struct. Biol.* **2014**, *186* (1), 195-203. DOI: 10.1016/j.jsb.2014.02.007.

(4) Zanghellini, A.; Jiang, L.; Wollacott, A. M.; Cheng, G.; Meiler, J.; Althoff, E. A.; Rothlisberger, D.; Baker, D. New algorithms and an in silico benchmark for computational enzyme design. *Protein Sci.* **2006**, *15* (12), 2785-2794. DOI: 10.1110/ps.062353106.

(5) Richter, F.; Leaver-Fay, A.; Khare, S. D.; Bjelic, S.; Baker, D. De novo enzyme design using Rosetta3. *Plos One* **2011**, *6* (5), e19230. DOI: 10.1371/journal.pone.0019230.

(6) Khatib, F.; Cooper, S.; Tyka, M. D.; Xu, K.; Makedon, I.; Popovic, Z.; Baker, D.; Players, F. Algorithm discovery by protein folding game players. *Proc. Natl. Acad. Sci. U. S. A.* **2011**, *108* (47), 18949-18953. DOI: 10.1073/pnas.1115898108.

(7) Mulligan, V. K. *Scorefunctions that work well with protein and non-protein residues and molecules*. Baker Laboratory, https://www.rosettacommons.org/docs/latest/rosetta_basics/scoring/NC-scorefunction-info#useful-terms-that-can-be-appended-to-scorefunctions_penalty-function-for-aspartimide-promoting-sequences-aspartimide_penalty

(8) Pace, C. N.; Vajdos, F.; Fee, L.; Grimsley, G.; Gray, T. How to measure and predict the molar absorption coefficient of a protein. *Protein Sci.* **1995**, *4* (11), 2411-2423. DOI: 10.1002/pro.5560041120.

(9) Strohalm, M.; Kavan, D.; Novak, P.; Volny, M.; Havlicek, V. mMass 3: a cross-platform software environment for precise analysis of mass spectrometric data. *Anal. Chem.* **2010**, *82* (11), 4648-4651. DOI: 10.1021/ac100818g.

(10) Greenfield, N. J. Using circular dichroism spectra to estimate protein secondary structure. *Nat. Protoc.* **2006**, *1* (6), 2876-2890. DOI: 10.1038/nprot.2006.202.

(11) Greenfield, N. J. Analysis of the kinetics of folding of proteins and peptides using circular dichroism. *Nat. Protoc.* **2006**, *1* (6), 2891-2899. DOI: 10.1038/nprot.2006.244.

(12) Kuzmic, P. Program DYNAFIT for the analysis of enzyme kinetic data: application to HIV proteinase. *Anal. Biochem.* **1996**, *237* (2), 260-273. DOI: 10.1006/abio.1996.0238.

(13) Kabsch, W. XDS. *Acta Crystallogr. D* **2010**, *66* (Pt 2), 125-132. DOI: 10.1107/S0907444909047337.

(14) Evans, P. R.; Murshudov, G. N. How good are my data and what is the resolution? *Acta Crystallogr. D* **2013**, *69* (Pt 7), 1204-1214. DOI: 10.1107/S0907444913000061.

(15) Winn, M. D.; Ballard, C. C.; Cowtan, K. D.; Dodson, E. J.; Emsley, P.; Evans, P. R.; Keegan, R. M.; Krissinel, E. B.; Leslie, A. G.; McCoy, A.; et al. Overview of the CCP4 suite and current developments. *Acta Crystallogr. D* **2011**, *67* (Pt 4), 235-242. DOI: 10.1107/S0907444910045749.

(16) Mirdita, M.; Schutze, K.; Moriwaki, Y.; Heo, L.; Ovchinnikov, S.; Steinegger, M. ColabFold: making protein folding accessible to all. *Nat. Methods* **2022**, *19* (6), 679-682. DOI: 10.1038/s41592-022-01488-1.

(17) McCoy, A. J.; Grosse-Kunstleve, R. W.; Adams, P. D.; Winn, M. D.; Storoni, L. C.; Read, R. J. Phaser crystallographic software. *J. Appl. Crystallogr.* **2007**, *40* (Pt 4), 658-674. DOI: 10.1107/S0021889807021206.

(18) Adams, P. D.; Afonine, P. V.; Bunkoczi, G.; Chen, V. B.; Davis, I. W.; Echols, N.; Headd, J. J.; Hung, L.-W.; Kapral, G. J.; Grosse-Kunstleve, R. W.; et al. PHENIX: a comprehensive Python-based system for macromolecular structure solution. *Acta Crystallogr. D* **2010**, *66* (2), 213-221. DOI: 10.1107/S0907444909052925.
(19) Emsley, P.; Lohkamp, B.; Scott, W. G.; Cowtan, K. Features and development of Coot. *Acta Crystallogr. D* **2010**, *66* (Pt 4), 486-501. DOI: 10.1107/S0907444910007493.
(20) Afonine, P. V.; Grosse-Kunstleve, R. W.; Echols, N.; Headd, J. J.; Moriarty, N. W.; Mustyakimov, M.; Terwilliger, T. C.; Urzhumtsev, A.; Zwart, P. H.; Adams, P. D. Towards automated crystallographic structure refinement with phenix.refine. *Acta Crystallogr. D* **2012**, *68* (Pt 4), 352-367. DOI: 10.1107/S0907444912001308.
(21) Chen, V. B.; Arendall, W. B., 3rd; Headd, J. J.; Keedy, D. A.; Immormino, R. M.; Kapral, G. J.; Murray, L. W.; Richardson, J. S.; Richardson, D. C. MolProbity: all-atom structure validation for macromolecular crystallography. *Acta Crystallogr. D* **2010**, *66* (Pt 1), 12-21. DOI: 10.1107/S0907444909042073.
(22) Sutherland, M. W.; Learmonth, B. A. The Tetrazolium Dyes MTS and XTT Provide New Quantitative Assays for Superoxide and Superoxide Dismutase. *Free Radical Res.* **1997**, *27* (3), 283-289. DOI: 10.3109/10715769709065766.
